# Adipocyte nuclei captured from VAT and SAT

**DOI:** 10.1186/s40608-016-0112-6

**Published:** 2016-07-19

**Authors:** Suresh Ambati, Ping Yu, Elizabeth C. McKinney, Muthugapatti K. Kandasamy, Diane Hartzell, Clifton A. Baile, Richard B. Meagher

**Affiliations:** Department of Genetics, University of Georgia, Athens, GA USA; Department of Foods and Nutrition, University of Georgia, Athens, GA USA; Department of Animal and Dairy Science, University of Georgia, Athens, GA USA

**Keywords:** Epigenetics, INTACT transgenic mouse, Visceral and subcutaneous adipose tissue, Cell-type specific

## Abstract

**Background:**

Obesity-related comorbidities are thought to result from the reprogramming of the epigenome in numerous tissues and cell types, and in particular, mature adipocytes within visceral and subcutaneous adipose tissue, VAT and SAT. The cell-type specific chromatin remodeling of mature adipocytes within VAT and SAT is poorly understood, in part, because of the difficulties of isolating and manipulating large fragile mature adipocyte cells from adipose tissues.

**Methods:**

We constructed MA-INTACT (Mature Adipocyte-Isolation of Nuclei TAgged in specific Cell Types) mice using the *adiponectin (ADIPOQ)* promoter (ADNp) to tag the surface of mature adipocyte nuclei with a reporter protein. The SUN1mRFP1Flag reporter is comprised of a fragment of the nuclear transmembrane protein SUN1, the fluorescent protein mRFP1, and three copies of the Flag epitope tag.

**Results:**

Mature adipocyte nuclei were rapidly and efficiently immuno-captured from VAT and SAT (MVA and MSA nuclei, respectively), of MA-INTACT mice. MVA and MSA nuclei contained 1,000 to 10,000-fold higher levels of adipocyte-specific transcripts, *ADIPOQ, PPARg2, EDNRB,* and *LEP,* relative to uncaptured nuclei, while the latter expressed higher levels of leukocyte and endothelial cell markers *IKZF1, RETN, SERPINF1, SERPINE1, ILF3, and TNFA.* MVA and MSA nuclei differentially expressed several factors linked to adipogenesis or obesity-related health risks including *CEBPA, KLF2, RETN, SERPINE1,* and *TNFA*. The various nuclear populations dramatically differentially expressed transcripts encoding chromatin remodeler proteins regulating DNA cytosine methylation and hydroxymethylation (*TETs, DNMTs, TDG, GADD45s*) and nucleosomal histone modification (*ARID1A, KAT2B, KDM4A, PRMT1, PRMT5, PAXIP1*). Remarkably, MSA and MVA nuclei expressed 200 to 1000-fold higher levels of thermogenic marker transcripts *PRDM16* and *UCP1*.

**Conclusions:**

The MA-INTACT mouse enables a simple way to perform cell-type specific analysis of highly purified mature adipocyte nuclei from VAT and SAT and increases the statistical significance of data collected on adipocytes. Isolated VAT and SAT adipocyte nuclei expressed distinct patterns of transcripts encoding chromatin remodeling factors and proteins relevant to diabetes, cardiovascular disease, and thermogenesis. The MA-INTACT mouse is an useful model to test the impact of caloric intake, dietary nutrients, exercise, and pharmaceuticals on the epigenome-induced health risks of obesity.

**Electronic supplementary material:**

The online version of this article (doi:10.1186/s40608-016-0112-6) contains supplementary material, which is available to authorized users.

## Introduction

Subcutaneous and visceral adipose tissues, SAT and VAT, become the dominant endocrine organs in obese individuals [1–3]. Increased VAT is linked to insulin resistance, diabetes, hypertension, atherosclerosis, and increased hepatic fat content [4, 5]. By contrast, obese SAT continues to respond better to insulin and is not as clearly linked to disease. Mature adipocytes (MAs) within SAT and VAT, MSAs and MVAs, respectively, have different developmental histories, distinct programs of gene expression, and dissimilar profiles of endocrine secretion [6–10]. MVAs and MSAs contribute distinctly to increased health risk due to altered metabolic properties and changes in adipokine secretion [11–14]. In general, when compared to tissue form normal weight individuals, obese VAT in particular and to a lesser extent obese SAT secrete harmfully higher levels of angiotensin, TNFα, IL1B, SERPINE1-1, and LEP as well as lower levels of generally beneficial IL10, ADIPOQ, omentin, APLN, and visfatin [15–19]. Therefore, VAT derived obese MVAs are more pathogenic [4]. Beige adipocytes in SAT, which are metabolically beneficial in normal weight individuals, become much less active with obesity and aging [20–22]. Not only does the number of MAs in VAT and SAT increase with obesity, but the cells themselves become significantly enlarged. Obese MAs promote inflammation, recruit macrophages, and predispose obese individuals to health risks, including the increased occurrence of cardiovascular disease, cancer, and diabetes [4, 23–25]. Hence, there has been intense interest in recent years in understanding the role of MAs in obesity-related comorbidities.

Epigenetics is by its origin the study of cell-type specific differences [26–31]. While cell type specificity may be the mantra of epigenetic analyses, few studies are performed at the cell-type specific level despite data showing the statistical significance of working at this level. As an example of the importance of performing cell-type specific analyses, among the seven major classes of peripheral blood leukocyte types in healthy human subjects, the two most similar (CD4 and CD8 T cells) and the two most dissimilar (CD8 and eosinophils) white blood cell types differ at 45,000 (9 %) and 190,000 (40 %), respectively, of 475,000 DNA 5´-methylcytosine (5mC) residues at informative CG dinucleotide sites [32]. Hence “*whole blood methylation results might be unintelligible*” because the data represent the weighted average of cell type differences. In addition, disease-related stress may cause the reprogramming of hundreds of 5mC sites within a single cell type [33, 34]. Studies of systemic lupus erythematosus (SLE) identified hundreds of 5mC sites in CD4+ T cells associated with the disease phenotype with median p values of ~10^−7^, while median *p* values of ~10^−3^ were found associated with SLE when DNA from the whole blood leukocyte fraction was examined [33, 35]. In short, the extra effort involved in examining enriched cell-type specific populations from a tissue may be expected to produce dramatically more meaningful epigenetic data [32].

While there is little reason to doubt that epigenetic reprogramming of obese adipocytes within their tissue context is an important component of disease risk [36–43], there are considerable challenges to performing cell-type specific epigenetic analysis of tissue-derived MAs. Adipose tissues comprise a complex mixture of cells including adipocytes, diverse endothelial cells and leukocytes, and various multipotent progenitor cell types. Obese SAT and VAT may have an additional bias in the weighted average of cell types, because the number of inflammatory leukocyte cell types may increase several-fold over that found in the tissue of normal weight individuals [44–46]. Another bias may be introduced during cell isolations. MAs are extremely large often ranging from 50 to 120 microns in diameter, generally having a single lipid droplet. They may be enzymatically dissociated and enriched by floatation away from other cell types [47], but they are still difficult to isolate and manipulate efficiently due to their fragile structures and tendency to lyse rapidly with handling or while sitting on ice. Even larger MA cell sizes are observed in obese VAT and SAT, which inherently exacerbates problems of cell manipulation. By contrast, various classes of multipotent and progenitor adipose tissue derived stem cells (ADSCs) and dedifferentiated adipocyte-derived progeny cells (DFAT cells) may be isolated intact and viable from SAT and VAT, in part, because they are small (15 to 25 microns) and relatively stable cells [48–51]. In short the physiology of adipocytes does not favor their isolation relative to other cells in VAT and SAT.

**Fig. 1:**
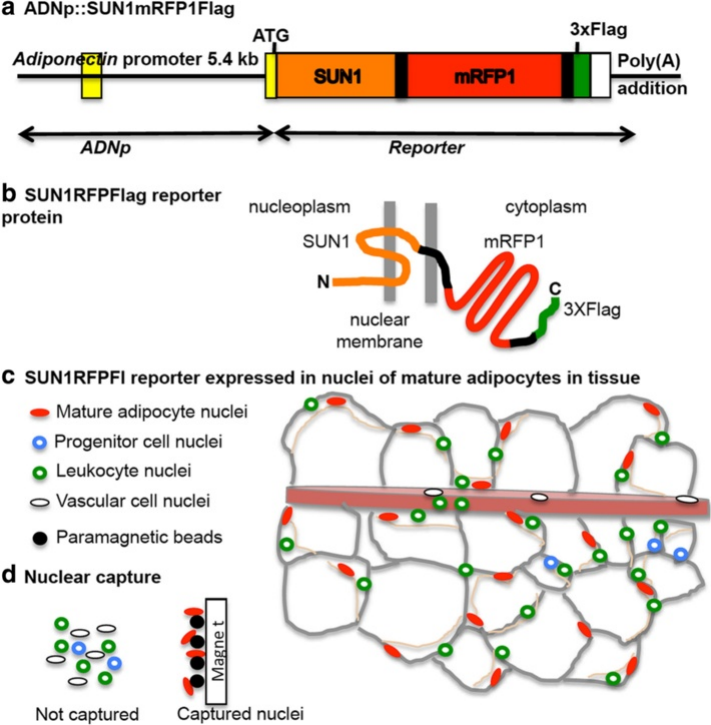
Strategy for implementing INTACT to capture adipocyte nuclei. **a** The *ADNp::SUN1mRFP1Flag* gene construct. This promoter is a truncated 5.4 kb version of the mouse adiponectin promoter (*ADNp*) modified slightly from one described previously [63]. This promoter was fused first to the N-terminal portion of mouse SUN1, then via a flexible linker peptide to the enhanced red fluorescent protein mRFP1, which was fused in frame through a linker peptide to a epitope Flag tag containing three distinct Flag sequences (3xFlag). The *ADIPOQ* promoter region contains a 5´ upsteam exon (yellow) and intron, a 5´ mRNA UTR, and the ATG start codon. The 3´ UTR and poly(A) addition site are from SV40 in the pcDNA3.1 vector. More detailed maps are shown in Additional file 1: Figure S1 and Additional file 3: Figure S3. **b** Proposed deposition of the SUN1mRFP1Flag protein tethered to the inner nuclear membrane and tagging the exterior of the nucleus. **c** Diagram of adipose tissue comprised of MAs expressing the SUN1mRFP1Flag tag and many non-target cell types (progenitor cells, leukocytes, endothelial cells). **d** Capture and enrichment of MA nuclei away from other cellular nuclei on paramagnetic beads labeled with anti-mRFP1, anti-GFP or anti-Flag. A total of four constructs designed from 3 nuclear transmembrane proteins were examined (Table 1)

Herein, addresses the problem of cell-type specific analysis of chromatin structure in MAs by applying INTACT technology (isolation of nuclei tagged in specific cell types) to MAs in a transgenic mouse model. INTACT has been used to isolate nuclei from specific cell types tagged in transgenic *Arabidopsis thaliana*, *Caenorhabditis elegans*, and *Drosophila melanogaster* [28, 52–54]. We developed a protocol that simplifies the isolation of cellular nuclei from VAT, SAT, and BAT. We screened for Nuclear membrane Targeted Fusion proteins (NTFs), which when expressed from an adiponectin derived promoter (ADNp), delivered both a fluorescent reporter and epitope tags to the exterior surface of MA nuclei. Our strategy for constructing a MA-INTACT mouse is outlined in Fig. 1. MA nuclei from transgenic mice expressing *ADNp::SUN1mRFP1Flag* are highly enriched after capture on immuno-paramagnetic beads. Captured and uncaptured classes of SAT and VAT nuclei differentially expressed many of the expected cell type markers, but expressed distinctly different chromatin remodeling factors and markers of thermogenesis.

## Materials and methods

### Reagents

Anti-RFP (ab34771), Anti-Flag (ab106259), anti-CD4 (ab846) antibodies were obtained from Abcam. Anti-mRFP1 rabbit polyclonal antibody (rab pAb) was prepared by Thermo Scientific Pierce Antibodies using their 2 Rabbit, 90 Day Protocol. We expressed the 672 bp *mRFP1* CDS with a C-Terminal 6xhis tag in *E. coli* from the PET15b vector. After IPTG induction the protein levels were very high and the cells turned red. The red protein was highly enriched on a column with nickel charged resin, Ni-NTA Superflow (Qiagen 30410). The IgG fraction of anti-mRFP1 antibody was enriched by ammonium sulfate fractionation [55] and dialyzed in phosphate buffered saline (10 mM H_2_NaPO_4_/HNa_2_PO4 pH = 7.4, 150 mM NaCl, 0.1 % sodium azide) for storage at 4 °C.

### NTF fusion proteins to tag adipocyte nuclei

An adiponectin promoted vector plasmid pADNpcDNA3.1 KanR was constructed to drive NTF expression in MAs (Additional file 1: Figures S1, Additional file 2: Figure S2). Four constructs were made from three NTFs with the potential to tag the surface of adipocyte nuclei with an epitope(s) to be used in immuno-capture experiments (Additional file 3: Figure S3, Additional file 4: Figure S4, Additional file 5: Figure S5, Additional file 6: Figure S6). All three proteins are anchored via N-terminal domains facing into the nucleoplasm and C-terminal domains facing outward toward the cytoplasm. Mouse SUN1 makes three passes through the inner nuclear membrane. One construct, *SUN1mRFP1Flag*, was made by fusing the N-terminus of mRFP1 and a triple Flag tag to the C-terminal end of a SUN1 N-terminal fragment (i.e., lacking the normal SUN1 C-terminus). Mouse Nesprin-3 (Nesp3) makes one pass through the outer nuclear membrane. Two constructs were made based on Nesp3. The first, *Nesp3mRFP1Flag*, is a simple fusion of mRFP1 and 3XFlag domain from *SUN1mRFP1Flag* to the C-terminus of an N-Terminal Nesp3 fragment (i.e., lacking the normal C-terminus of Nesp3). Because the C-terminus of Nesp3 has been implicated in its localization we made a second construct. In *Nesp3mRFP1Nesp*, we added the C-terminal domain of Nesp3 to the end of mRFP1, replacing the 3XFlag sequence in Nesp3mRFP1Flag. In each of the three above constructs, (GlySer)_4-5_Gly linkers separate the different domains. *Arabidopsis* RANGAP1 associates with the cytoplasmic side of the nuclear pore complex. We used the same RANGAP1GFP reporter sequence that was used in the first successful report of INTACT technology in *Arabidopsis* [28]. All four constructs were inserted into the adiponectin expression vector. Construct sequences will be available at NCBI’s sequence repository (DDBJ/EMBL/Genbank - INSDC) once this manuscript is accepted for publication.

### Cell culture

Mouse embryo fibroblast cell line (3T3-L1, ATCC #CL-173) was purchased from American Type Culture Collection (Manassas, VA). Cells were cultured in DMEM (Invitrogen #12800017) containing 10 % bovine calf serum (Life Technologies, #16170078), 1 % penicillin-streptomycin-glutamine (Life Technologies, #10378016) and 1 % (v/v) 100 mM sodium pyruvate (Life Technologies, #11360070) at 37 °C in 95 % air-5 % CO_2_.

### Nucleofection

3T3-L1 preadipocytes (2 × 10^6^ cells) were nucleofected with each construct (0.1, 0.5, 1, and 2 ug DNA) using the Amaxa Cell Line Nucleofector Kit V (Lonza #VCA-1003). Each nucleofection was seeded into a 6 well microtiter plate in calf serum medium and expanded for 6 days with700 ug of neomycin G418 (Invitrogen #10131035). Cells were transferred to 50 mm petri plates and grown. Two days after reaching confluence, cells were induced to differentiate for two days with DMEM media containing 10 % fetal bovine serum (FBS) (Life Technologies, #10437028), 0.5 μM 3-isobutyl-1-methylxanthine (IBMX, Sigma #I5879), 1 μM dexamethasone (Sigma #D4902) and 167 nM insulin (Sigma #I-6634). Cells were maintained for the next two days in DMEM medium containing 10 % FBS and 167 nM insulin. Cells were cultured for an additional 4 days in 10 % FBS/DMEM medium, during which time they differentiated into MAs filled with lipid-droplets. MAs were maintained in 10 % FBS/DMEM medium and analyzed daily for green or red fluorescent protein expression by fluorescence microscopy (FM) on Nikon (Eclipse TE2000-S) inverted microscope.

#### Transgenic MA-INTACT mice expressing SUN1mRFP1Flag nuclear tag in MAs

A 12.4 kb NotI fragment from our plasmid vector containing *ADNp::SUN1mRFP1Flag* reporter sequence (Additional file 1: Figure S1 & Additional file 3: Figure S3) was used to construct transgenic (Tg) mice in the C57 Black 6 (C57BL/6 N) background. Among the mice identified with the randomly integrated transgene, four mice expressed *SUN1mRFP1Flag* mRNA in tail snip mRNA preparations (Additional file 7: Figure S8). We identified two founding mice, C023 and D025 (C and D lines), whose offspring expressed significant amounts of the *SUN1mRFP1Flag* mRNA in BAT, VAT, and SAT. We generated two inbred mouse colonies, C023 and D025, homozygous for the transgene. Both appear healthy and long-lived. The studies reported herein represent data from hemizygous F2 and F3 generation animals used in breeding.

### Enrichment of nuclei

The following rapid protocol for isolating adipose tissue cellular nuclei or nuclei from transfected 3T3-L1 cells follows closely to that which we published recently for brain cell nuclei [30] (Additional file 8: Figure S7). Freshly dissected and minced mouse tissue or 3T3-L1 adipocytes were treated for 15 min to 1 hr in four volumes (w/v) of 0.3SPBSTA (0.3 M Sucrose, 20 mM KH2PO4, 20 mM Na_2_HPO_4_, pH = 7.2, 137 mM NaCl, 3.0 mM KCl, 0.1 % Triton X 100, 0.1 % sodium azide), plus 3.7 % freshly added formalin. Tissue was washed twice with phosphate buffered saline. Tissue samples (100 to 200 mg) were homogenized by hand in a 1.5 ml microfuge tube with a plastic pestle for 1 min in 6 volumes of 0.3SPBSTA. The homogenate was filtered through small pieces (5x5 cm sq) of Miracloth (Calbiochem, #475855) suspended loosely over a small funnel. This prevented nuclei from being trapped with large pieces of cytoplasm during the subsequent centrifugation and increased the yield of nuclei several fold. Nuclei were centrifuged through a sucrose cushion (1.4 M sucrose, in 1.4SPBSTA) in a swinging bucket rotor in a refrigerated centrifuge (4 °C) at 3,000xg for 15 min. The nuclear pellet was gently re-suspended in 10 volumes of 0.3 M SPBSTA Microscopic assays using differential interference phase microscopy (DIC) combined with DAPI staining for DNA suggested that the nuclei were approximately 80 % pure, relative to visible debris that did not stain with DAPI (Additional file 8: Figure S7). All reagents were purchased from Thermo Fisher (Waltham, MA), unless stated otherwise.

### Immuno-capture of nuclei

Anti-RFP, anti-FLAG, anti-CD4, anti-CD8, anti-mRFP1 antibodies were coupled to 2.8 micron diameter protein G supraparamagnetic Dynabeads (ThermoFischer Scientific, #10004D). Bead protocols including the coupling were handled as per the manufacturer’s instructions. Briefly, antibody coupled beads were incubated with nuclei prepared from SUN1mRFP1Flag nucleofected mice and differentiated 3T3-L1 adipocytes and washed extensively to remove uncaptured nuclei. The first uncaptured fraction was assayed in parallel. During the wash steps the bead-bound nuclei were repeatedly pulled to the side of 1.5 ml microcentrifuge tubes for 1 min using neodymium magnets (DynaMag™-2 Magnet rack, #12321D, ThermoFischer Scientific).

### Assays of nuclear transcript levels

Relative quantities (RQ) of nuclear RNA levels from whole VAT, SAT, and BAT tissue, total isolated nuclei, captured and uncaptured nuclei were quantified with qRT-PCR assays. The RQ of transcripts were calculated based on the dCT method including the standard deviation from the mean (Livak and Schmittgen, 2001). Primers are listed in Additional file 9: Table S1. *SDHA* mRNA was used as the endogenous control to calculate RQ, because in repeated assays its levels, based on cycle threshold values (CTs), varied less than 1- to 1.8-fold relative to input cDNA among various samples of captured and uncaptured nuclear cDNA preparations, similar to what we described for fractionated brain cellular nuclei [56]. Based on CT values, *18S rRNA* and beta*-actin* [57] were also reasonably equivalently expressed among cDNA samples and would have been reasonable endogenous controls, but their CT values varied slightly more than *SDHA* among nuclear RNA samples.

RNA from fresh or formalin fixed nuclei was prepared using an RNeasy FFPE kit (Qiagen #73504). If formalin treated nuclei were used, a heat treatment of 90 °C for 1 h was included, after the proteinase K digestion in PKD buffer to hydrolyze off the formalin [56]. The heat-accelerated hydrolysis of formalin crosslinks is well established (Fraenkel-Conrat and Olcott, 1948). RNA was quantified using a Qubit RNA Assay Kit (Life Technologies, #Q32855). The yield of RNA from 100,000 fixed sorted nuclei varied from 0.5 to 1 μg. 500 ng samples of RNA were reverse transcribed (RT) into cDNA using qScript cDNA SuperMix (Quanta BioSciences, #95048). After RT,0.5 μl of RNase H at 5 units/μl (New England Biolabs, #M0297S) was added to the 20 μl reaction and incubated at 37 °C for 20 min. cDNA yield was quantified using the Qubit ssDNA (single strand DNA Assay Kit #Q10212). 5 ng of cDNA was used per reaction in qRT-PCR assays. Primers are described in Additional file 9: Table S1. Each assay was run in triplicate and the standard error calculated to give the error bars shown. Three biological replicates gave similar results.

### Statistical analysis

The mean ± standard error of the mean (SEM) is presented for various bar graph data. The qRT-PCR data were further analyzed by one-way ANOVA with post hoc Tukey’s HSD test using Statistica software 6.1 (StatSoft; Tulsa, OK, USA). Noteworthy statistical comparisons are indicted in the figures with * for *p* < 0.05, ** for *p* < 0.01, and *** for *p* < 0.001 and some p values are also stated in the text.

## Results

### A mature-adipocyte-specific expression vector

Adiponectin (ADIPOQ) is one of the most abundant adipose-tissue derived hormones and has multiple anti-metabolic and anti-inflammatory properties [58]. ADIPOQ is expressed in MAs from SAT and VAT and a subset of non-thermogenic adipocytes from BAT, but is not significantly expressed in other adipose tissue-derived cell types including endothelial cells, leukocytes, and preadipocytes [59–61]. ADIPOQ is expressed in a few other cell types in the brain [62] that will not affect our targeting of MAs for capture from adipose tissue. *ADIPOQ* promoter driven transcript expression is more specific for MAs than is expression from the promoters of two other commonly used marker genes expressed in adipocytes *PPARg* and *FABP4* [63]. Therefore, we used an *ADIPOQ* promoter to express INTACT constructs that might tag MAs.

We engineered an mouse *adiponectin* promoter (ADNp) expression vector (ADNppcDNA3.1) based on a truncated 5.4 kb version of the full length promoter reported previously to retain its adipocyte cell-type specificity [63]. ADNp was incorporated into the backbone of the commonly used expression vector pcDNA3.1 (NeoR) (Materials and Methods, Additional file 1: Figure S1). To test the specificity and transcriptional activity of this vector, we inserted the coding sequence (CDS) of a red fluorescent protein, mRFP1, to make a *ADNp::mRFP1* reporter (Additional file 2: Figure S2) [64]. Assaying red fluorescent protein (RFP) avoids the low level of endogenous green fluorescence observed in some adipose tissues. Relative to other RFPs, mRFP1 has the advantages of a modestly high fluorescence extinction coefficient and a rapid protein maturation rate. Furthermore, mRFP1 does not form a multimeric cytoplasmic aggregate as do many other fluorescent proteins, which might interfere with its efficient localization to the nuclear membrane.

**Fig. 2:**
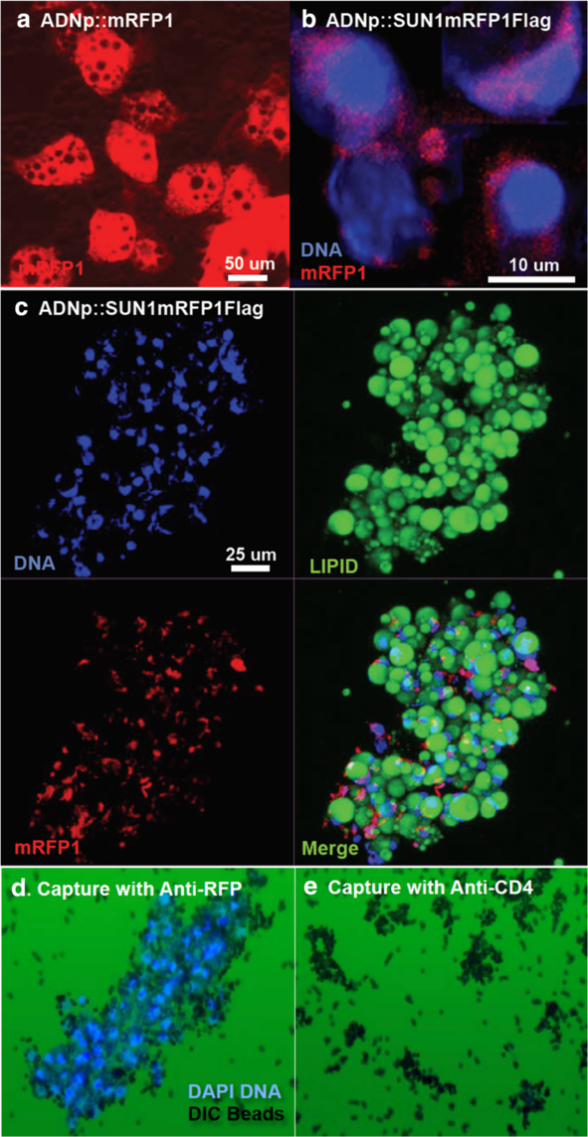
Expression of reporters and capture SUN1mRFP1Flag tagged nuclei from mature 3T3-L1 adipocytes. **a** Six days after *ADNp::mRFP1* nucleofected 3T3-L1 preadipocytes were induced to differentiate into MAs strong cytoplasmic fluorescence of the mRFP1 reporter was detected (see Table 1, Additional file 2: Figure S2). Pre-adipocytes did not have detectable fluorescence. This result shows the promoter vector was specifically expressed in MAs. **b** mRFP1 fluorescence from the ADNp::SUN1mRFP1Flag reporter on the surface of differentiated 3T3-L1 nuclei selected from c. **c** Expression of the ADNp::SUN1mRFP1Flag reporter in MAs 10 days after induction of nucleofected 3T3-L1 preadipocytes (DAPI stained DNA (*blue*), lipid rich oil droplets detected with Lipotox Green (*green*), enhanced red fluorescent protein mRFP1 (*red*). The lower right hand panel shows the merged fluorescence image. mRFP1 fluorescence is associated with the nuclei. It should be noted that spherical oil droplets often produce an optical distortion of adjacent nuclei. **d** Adipocyte nuclei from mature 3T3-L1 cells expressing the SUN1mRFP1Flag reporter were captured with Protein-G Dynabeads (2.8 μm diameter) pre-adsorbed with anti-GFP antibody. **e** A negative control capture performed in parallel using Protein-G Dynabeads pre-adsorbed with anti-CD4 T cell specific antibody did not capture nuclei

*ADNp::mRFP1* was nucleofected into 3T3-L1 preadipocytes. After 6 days of G418 selection for the linked neomycin resistance marker NeoR, cells were transferred to adipocyte differentiation medium. No red fluorescence was observed in nucleofected preadipocytes during cellular expansion prior to differentiation. mRFP1 fluorescence was detected in some cells 3 or 4 days after initiating differentiation and red fluorescence was very strong in many clusters of cells by 5 to 7 days (Table 1). We did not observe an obvious change in overall fluorescence intensity even after 14 days, except that cells filled with oil bodies showed little fluorescence. As shown in Fig. 2a, mRFP1 fluorescence was observed surrounding newly formed oil bodies within MAs. Hence, the ADNp promoter in ADNppcDNA3.1 exhibited sufficient specificity and strength to warrant its use in tagging MA nuclei.

**Table 1:** Four constructs were tested for fluorescent reporter expression during the differentiation of 3T3-L1 preadipocytes

^a^ADNp::promoted reporter protein	Day 4	Day 5	Day 6	Day 7	Day 10	Day 14
mRFP1	+	++	+++	+++	+++	+++
SUN1mRFP1Flag	ND	ND	ND	+	+++	+++
Nesp3mRFP1	ND	ND	ND	ND	ND	+
Nesp3mRFP1Nesp	ND	ND	ND	+	++	++
RANGAP1GFP	ND	ND	ND	ND	+	++

*ND* not detected. Standard

^a^The time (days) after induction to differentiate into MAs when the fluorescent protein was detected is indicated. Preadipocytes were nucleofected with five adiponectin promoted reporters, one for cytoplasmic expression (mRFP1) to test the vector pADNpcDNA3.1 KanR and four others targeting nuclei and designed to implement INTACT technology

### Screening nuclear targeted fusion proteins for an effective INTACT construct

Little is known about the expression and molecular conformation of nuclear transmembrane proteins specific for MAs. Therefore, we tested the ability of portions of three nuclear transmembrane proteins that have been characterized in other cell types and organisms, SUN1, Nesprin 3, and RANGAP1, to target fluorescent reporters to the surface of adipocytes. Four distinct constructs based on these three genes were cloned into the multi-linker in ADNppcDNA3.1, replacing the mRFP1 gene in ADNp::mRFP1 (Table 1, Additional file 3: Figure S3, Additional file 4: Figure S4, Additional file 5: Figure S5, Additional file 6: Figure S6).

3T3-L1 preadipocyte cultures were nucleofected with each construct, subjected to neomycyin G418 selection and expansion for 6 days, and then differentiated into MAs. Live cells in culture dishes were screened by fluorescence microscopy for mRFP1 red or GFP green fluorescence associated with each construct. No fluorescence was detected prior to differentiation for any of the constructs, confirming again that the 5.4 kb ADNp promoter utilized was not leaky in preadipocytes. While all reporters fluorescently tagged MA nuclei, they varied widely in both the timing of first detection and the strength of nuclear fluorescence (Table 1). SUN1mRFP1Flag and Nesp3mRFP1Nesp proteins were both detected by day 7, after initiating differentiation, but the red fluorescence from the SUN1 based construct was considerably stronger than for the Nesprin 3 based construct. It took a few more days before we first visualized the red and green fluorescent reporters associated with the Nesp3mRFP1 or RANGAP1GFP constructs, respectively. Figure 2c shows a field of differentiated 3T3-L1 adipocytes nucleofected with ADNp::SUN1mRFP1Flag and photographed for the red fluorescence of mRFP1 portion of the fusion protein, green fluorescence of neutral lipids stained with Lipotox Green, and the blue fluorescence of DNA stained with DAPI. Nucleofection of 3T3-L1 preadipocytes is very efficient and after a few days of G418 enrichment for transfected preadipocytes nearly every nucleus in the field (~90 %) of MAs showed associated mRFP1 expression. We found the red fluorescence of nuclei from SUN1mRFP1Flag was not symmetrically distributed over the surface of nuclei and was often skewed to one side relative to DAPI staining of DNA (Fig. 2b). Based on these promising results we focused on the ADNp::SUN1mRFP1Flag construct.

### Capture of adipocyte nuclei

The asymmetric distribution of the nuclear tag should not confound our ability to capture nuclei. Nuclei were purified from mature 3T3-L1 adipocytes 14 days after initiating the differentiation following the protocol outlined in Additional file 8: Figure S7A. Figure 2d shows nuclei captured with anti-RFP rabbit pAb antibody coupled to protein G paramagnetic Dynabeads. Nearly all the mature 3T3-L1 adipocyte nuclei were captured. A negative control performed with Anti-CD4 antibody did not capture a significant number of nuclei (Fig. 2e), nor did other unrelated antibodies. Subsequent assays with commercial anti-Flag mouse monoclonal antibody and anti-mRFP1 rabbit polyclonal antibodies prepared later for this study both efficiently captured tagged 3T3-L1 adipocyte nuclei (not shown). Although the efficiency of capture was not quantified in detail, we did not detect any obvious difference in the capture with the three antigen tag-specific antibodies tested (anti-GFP, anti-mRFP1, anti-Flag), suggesting that the nuclear immunocapture technology is relatively robust.

### Capturing MA nuclei from transgenic MA-INTACT mice

**Fig. 3:**
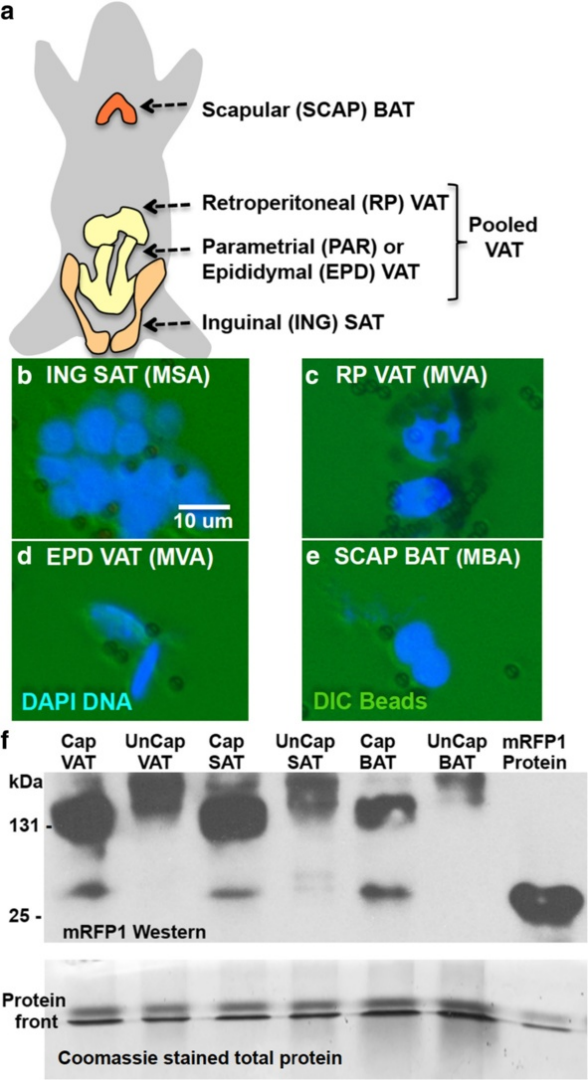
Mature MA-INTACT mouse adipocyte nuclei expressed *SUN1mRFP1Flag* RNA in BAT, VAT, and SAT allowing adipocyte nuclei to be captured on Immuno-paramagnetic beads. **a** Illustration of mouse fat deposits examined. **b**, **c**, **d**, **e**. Nuclei were efficiently captured from crude nuclear preparations prepared from inguinal SAT (**b**) retroperitioneal VAT (**c**) epididymal VAT (**d**) and scapular BAT (**e**). Protein G Dynabeads pre-adsorbed to rabbit anti-mRFP1 polyclonal antibody were used in these capture experiments. The clumping of nuclei occurs during successive rounds of washing and capture. Negative control antibodies did not capture significant numbers of nuclei (not shown). **f** A Western blot (*top panel*) showed that preparations of captured VAT, SAT, and BAT nuclei expressed the 131 kDa SUN1mRFP1Flag reporter protein, while uncaptured nuclei did not. In house prepared anti-mRFP1 rabbit pAb detected the mRFP1 domain. Purified 25 kDa mRFP1 was run as a positive control. A loading control (*bottom panel*) showed the approximately equal loading of nuclear proteins and an mRFP1 standard. The loading control samples were run for a short time (20 min) on an SDS PAGE system and protein front stained with Coomassie

MA-INTACT transgenic C57BL/6 mice were generated expressing the *ADNp::SUN1mRFP1Flag* transgene and the SUN1mRFP1Flag reporter in VAT, SAT, and BAT (see Materials and Methods). Two independent lines of mice, MA-INTACT-C and MA-INTACT-D derived from independent transformation events were developed. The C and D lines were indistinguishable from C57BL/6 mice in size and longevity. The breeding of these lines is described in Additional file 7: Figure S8. Three- to four-month-old MA-INTACT mice were sacrificed. Retroperitoneal (RP) and epididymal (EP) VAT, inguinal SAT, and scapular BAT (Fig. 3) were harvested. The tissues were immediately fixed in formalin to preserve chromatin structures, and nuclei were prepared as described in Materials and Methods and Additional file 8: Figure S7. Combined DIC and fluorescence microscopy of DAPI stained nuclei show these nuclear preparations were relatively free of contamination with cell debris (Figure S7C). Furthermore, Western blots immunostained for histone H3 showed that nuclei prepared from BAT, SAT, and VAT were highly enriched for this nuclear protein, relative to the amount of H3 in total protein extracts from these three tissues (Figure S7D).

The reporter tagged subsets of MA nuclei (MVA, MSA, and MBA) nuclei were captured using anti-mRFP1 rabbit polyclonal antibody pre-bound to protein G Dynabeads (Fig. 3b-e). The Western immune-blot probed with antibody to mRFP1 in Fig. 3f shows that 131 kDa SUN1mRFP1Flag fusion protein was associated with captured MA nuclei in mouse VAT, SAT, and BAT, but not with uncaptured nuclei. It appears a small amount of approximately 25 kDa mRFP1-immunoreactive protein the same size as mRFP1 itself (Right side of blot) may have been released by proteolytic degredation. Yields of nuclei from tissue and captured nuclei from 3-month-old mice are reported in Table 2. In repeated experiments approximately 45 %, 50 %, and 64 % of the total nuclei were captured from SAT, VAT, and BAT, respectively (Table 2). Similar proportions of adipocyte nuclei were captured from 7-month-old mice.

**Table 2:** Recovery of adipocyte nuclei captured on immuno-paramagnetic beads from VAT, SAT, and BAT dissected from the MA-INTACT mouse

Tissue	Tissue weight (ave.)	# nuclei recovered (ave.)/nuclei/mg	# adipocyte nuclei captured ave.^a^/% of total
VAT	370 ± mg	93,000±/251±	41,000/44.8 ± 6 %
SAT	160 ± mg	94,000±/587±	48,000/49.5 ± 3 %
BAT	85 ± mg	44,000±/518±	30,000/63.5 ± 8 %

^a^Standard errors computed from 5 capture experiments on 5 separate 3-month-old MA-INTACT mice using rabbit anti-mRFP1 polyclonal antibodies on 2.8 micron protein-G coupled paramagnetic Dynabeads. Similar, results were obtained when anti-RFP and anti-Flag antibodies were used as capture reagents

### Gene expression in captured SAT and VAT nuclei

We quantified transcript levels in cDNA prepared from nuclear RNA from SAT and VAT using qRT-PCR analysis. It is worth mentioning at this point that nuclear transcript levels strongly correlate with cytoplasmic mRNA levels for the vast majority of transcripts [28].

#### Transcripts enriched in adipocyte and non-adipocyte cell types in SAT and VAT

**Fig. 4:**
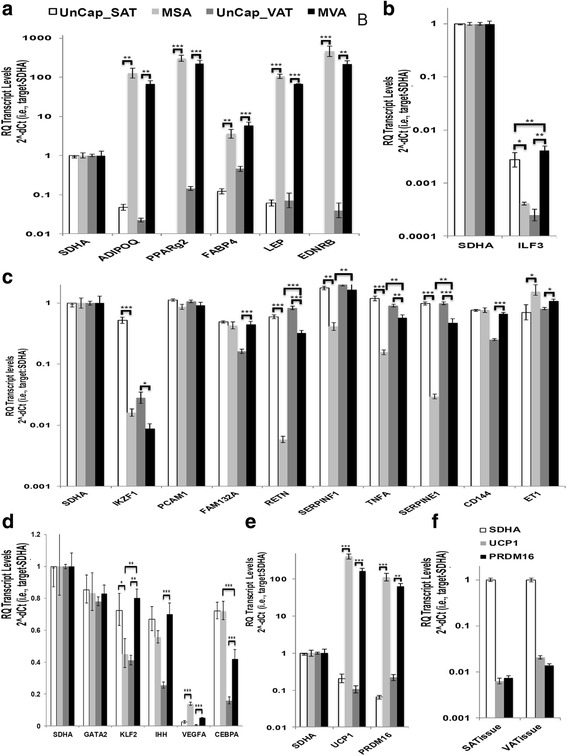
Expression of cell-type specific transcripts in MA nuclei captured from VAT and SAT. qRT-PCR analysis was performed on cDNA prepared from captured adipocyte nuclei from VAT (MVA) and SAT (MSA) and uncaptured nuclei (UnCap-VAT and -SAT). Nuclear mRNAs were assayed for transcripts encoding (**a**) *MA* markers, (**b**, **c**, **d**) potential markers for leukocytes, endothelial cells, and progenitor cells, (**e**) markers of thermogenic beige/bright adipocytes. **f** The levels of *PRDM16* and *UCP1* transcripts in whole VAT and SAT tissue were also assayed. *SDHA* was used as the endogenous control as it was nearly equivalently expressed in all samples relative to the amount of input cDNA. Bar graphs show the Mean ± SEM with *p* < 0.05 *, *p* < 0.01 **, *p* < 0.001 *** indicated for selected comparisons

The full names of marker transcripts are given in Abbreviations. *SDHA* was determined to be nearly equivalently expressed among captured (MSA, MVA) and uncaptured (Uncap) SAT and VAT nuclear RNA preparations based on equivalent cDNA input into qRT-PCR reactions, and therefore, *SDHA* was used as the endogenous control and set to one in calculations of Relative Quantity (see Materials and Methods. The RP and EP VAT RNA preparations from individual mice were combined to increase the amount of VAT RNA and simplify the analysis. While few marker transcripts are absolutely specific for a single cell type, transcripts expected to be highly enriched in mature white adipocytes relative to preadipocytes, leukocytes and/or endothelial cells were assayed first. Captured MSA and/or MVA nuclei expressed 10- to 10,000-fold higher levels of *ADIPOQ, PPARg2, FABP4, LEP, and EDNRB* transcripts*,* relative to Uncap-SAT and Uncap-VAT nuclei (Fig. 4a, *p* < 0.01 or 0.001)*. FABP4* is expressed at low levels in leukocytes [65]. Therefore, *FABP4* expression in uncaptured leukocyte nuclei may account for this marker transcript being the least enriched in the captured adipocyte nuclei. Transcripts encoding the adipocyte marker *FAM132A (adipolin)* were significantly (*p* < 0.001) more highly expressed in Cap-VAT nuclei than Uncap-VAT, but there was no difference between the two fractions of SAT nuclei (Fig. 4b). Hence, it appears that nuclear capture, based on expression of the SUN1mRFP1Flag reporter, efficiently enriches for MA nuclei. Second, transcripts with specificity for non-adipocyte cell types were assayed. Uncap VAT and/or Uncap-SAT expressed 2- to 100-fold higher levels of transcripts encoding most factors with specificity for leukocytes (*IKZF1*, *RETN, ILF3),* endothelial cells (*RETN, SERPINE1 (PAI1), SERPINF1 (PEDF), TNFA, CD144),* and progenitor cells *(CD34, KLF2)* relative to the levels in Cap-MSA and Cap-MVA nuclei (Fig. 4b, c, d). The majority of these differences were statistically significant (*p* < 0.01 or *p* < 0.001). In short, the Uncap-SAT and -VAT nuclear fractions appear to represent the expected enrichment of a mixture of non-adipocyte cell types. However, inconsistent with these data, the endothelial marker transcripts *ET1, VEGFA* and *CD144*, the leukocyte marker *IFL3,* and the progenitor cell markers *IHH* and *KLF2* were found at 2- to 10-fold higher levels in Cap-MVA nuclear fraction rather than Uncap-VAT fraction (*p* < 0.05 to *p* < 0.001).

#### Distinctions between captured MSA and MVA nuclei

**Fig. 5:**
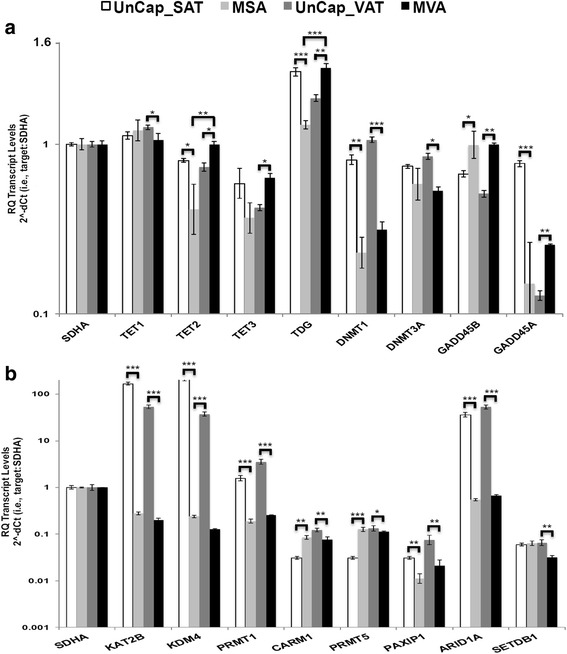
Expression of transcripts encoding chromatin remodeling factors in MA nuclei captured from VAT and SAT. **a** Transcripts encoding chromatin-remodeling proteins involved in the turnover cycle for DNA cytosine modification. **b** Transcripts encoding chromatin-remodeling proteins involved in nucleosomal histone modification and movement. For details see Fig. 4

Comparsions of the SAT and VAT transcriptomes from normal and/or obese subjects reveal the coexpression of approximately 90 % of transcripts identified and distinct expression of a remaining substantial subset [10, 66, 67]. We found that captured adipocyte nuclei from VAT and SAT (MVA and MSA nuclei) expressed similar levels of 31 of the 39 transcripts we assayed, but significantly differentially expressed the other eight (e.g., *CEBPA, ILF3, KLF2, RETN, SERPINF1, SERPINE1, TDG, TNFA*) (Figs. 4 and 5). *CEBPA, KLF2, RETN, SERPINE1* and *TNFA* have reported activities particular to adipocytes or obesity-related diseases. CEBPA is a transcription factor that assists with the differentiation of adipocytes [68]. We found 40 % lower levels of *CEBPA* in MVA than MSA nuclei (*p* < 0.001), similar to the results reported in a comparison of VAT and SAT mRNAs in relatively healthy obese men [66]. This same previous study showed higher levels of *VEGF* and *LEP* in VAT than SAT, but we did not observe such differences between MVA and MSA nuclear RNAs. KLF2 acts to suppress adipogenesis [69]. Thus, the two-fold higher levels of *KLF2* we found in MVA nuclei relative to MSA nuclei (*p* < 0.01) may reduce the relative levels of adipogenesis in VAT. TNFA is a cytokine secreted by macrophages linked to insulin resistance and diabetes that is also known to activate preadipocyte genes and inhibit adipogenesis [70, 71]. Most studies examine adipocytes exposed to exogenous TNFA, yet we observed substantial expression of endogenous *TNFA* transcripts in both MVA and MSA nuclei. Further, MVAs expressed several times more *TNFA* than MSAs (*p* < 0.01), again favoring reduced adipogenesis in VAT. Hence, differences in *CEBPA*, *KLF2* and *TNFA* transcripts all favor reduced adipogenesis VAT relative to SAT. We likely detected and resolved the differential expression of these genes between MSAs and MVAs, because their expression levels were not assayed among the transcripts in mixture of other adipose tissue cell types [10, 66, 67].

A few other transcripts differentially expressed in MVAs and MSAs may be linked to obesity health risk. Serum levels of the adipokine RETN (resistin) positively correlate with mortality risk from diabetes and cardiovascular disease [72, 73]. Captured MVA nuclei expressed 40-fold higher levels of *RETN* transcripts than MSA nuclei (*p* < 0.001). Serpins are protease inhibitors that unfold and lead to the degradation of their target proteases, and hence, play important roles in inflammation and cell death. A systems biology network analysis of the plasma proteome expressed in obese diabetic patients relative to normal healthy subjects found SERPINE1 at more central nodes of networks linked to other proteins with roles in obesity, diabetes, and cardiovascular disease, than any other plasma protein [74]. Transcripts for SERPINE1 and its distant sequence homolog SERPINF1 were 20-fold and five-fold, respectively, more highly expressed in MVA than MSA nuclei (*p* < 0.01). These transcript differences support the potential negative health outcome of having excess VAT.

Considering that little is known about the differentiation of white adipocytes into beige adipocytes within SAT and VAT, we assayed two factors normally associated the browning of adipose tissue and heat generating activity (Fig. 4e). PRDM16 is a transcription factor essential to brown/beige adipose tissue development and UCP1 is a nuclear encoded uncoupling protein 1 that generates heat in the mitochondria of brown and beige adipocytes. *PRDM16* and *UCP1* transcripts were significantly upregulated (200- to 1,000-fold) in Cap-MSA and Cap-MVA nuclei compared to uncaptured nuclei (*p* < 0.01 or 0.001) and 10,000-fold higher than they were expressed in their parent SAT and VAT tissues (Fig. 4f).

#### Captured BAT nuclei

SUN1mRFP1Flag tagged nuclei were captured from BAT as shown in Fig. 3f and quantified in Table 2. However, adiponectin (ADIPOQ) expression suppresses the thermogenic program in brown adipocytes [61] and reduces expression of UCP1, the main uncoupling protein that generates heat in brown adipocytes. Therefore, the *ADIPOQ* promoter driven reporter *ADNp::SUNmRFP1Flag* is not appropriate to tag thermogenic brown adipocytes. The results from our assaying a battery of cell type-specific and function-specific transcripts by qRT-PCR (Additional file 10: Figure S9) do not identify the captured nuclei as representing a single recognizable cell type. As might be expected from the inverse relationship between *ADIPOQ* and *UCP1*, uncaptured BAT nuclei expressed several fold higher levels of transcripts encoding *UCP1* and *APLN*[75] (*p* < 0.01), when compared to captured BAT adipocyte nuclei. APL is an adipocyte specific hormone that promotes the metabolic activity of brown adipocytes [75]. By contrast, captured MBA nuclei were enriched for some adipocyte enriched marker transcripts *FAM132A (adipolin), LEP* [76], *PAX3* [77] *RETN,* and *SERPINF1* [78] (*p* < 0.05 to 0.001), while neither nuclear population was enriched for other transcripts that might be considered adipocyte-enriched *(*e.g.*, PRDM16, PPARg2, FABP4, ZIC1)*. In addition, captured BAT nuclei were enriched for progenitor cell transcripts *CD34, GATA2, KLF2*, and *IHH* (*p* < 0.05 to 0.01). Finally, and adding to the complexity of this result, endogenous *ADIPOQ* transcripts were equivalently expressed in captured and uncaptured nuclei. We speculate that the *ADIPOQ* promoter driven reporter tagged multiple cell types in BAT.

#### Transcripts encoding chromatin remodeling proteins

We assayed transcript encoding two classes of chromatin remodeling factors. First, we examined mRNAs for factors involved in the cycle of DNA cytosine modification and turnover, because of their role in adipogenesis. DNA cytosine methylation in preadipocytes during the contact inhibition stage of growth appears essential to licensing their ability to differentiate at a later stage into mature lipid containing adipocytes [79]. During the execution of adipogenesis cytosine demethylation of PPARg2 promoter is essential for PPARg2 expression [80]. By contrast, the gene for the bromodomain suppressor of adipogenesis *BDR2* becomes increasingly methylated and silenced during adipogenesis [81]. Our analysis included two DNA cytosine methyltransferases *DNMT1* and *DNMT3A* that methylate cytosine (C) to 5-methylctosine (5mC), three 5mC dioxygenases (*TET1, 2, 3*) that oxidize 5mC to 5-hydroxymethylcytosine (5hmC) and subsequently to more oxidized forms (5caC and 5fC), thymine glycosylase *TDG* that removes 5caC and 5fC to generate an abasic site, and repair enzymes *GADD45A* and *GADD45B* that restore C residues to the abasic sites (Fig. 5a). *TET2, TET3, TDG, GADD45A* and *GADD45B* transcripts were at higher levels in Cap-MVA nuclei than Uncap-VAT nuclei (*p* < 0.05 to 0.01). The encoded proteins are all involved in removal of 5mC and cycling back to C, suggesting that the cycle of cytosine modification might run faster in VAT-derived adipocytes. In contrast, *TET2, TDG, DNMT1*, and *GADD45A* transcripts were at higher levels in Uncap-SAT than Cap-MSA nuclei (*p* < 0.05 to 0001). TDG (thymine DNA deglycosidase) participates in the turnover of DNA 5´-methylcytosine and in the repair of DNA in terminally differentiated cells. TDG is expressed in human adipose tissue derived mesenchymal stem cells and [82]. Its 4-fold higher expression in MVA than MSA nuclei might represent regulatory or repair functions. In short the transcript expression for factors involved in cytosine modification and turnover are distinctly differentially expressed in SAT- and VAT-derived nuclear populations, which should impact adipogenesis.

Second, we examined factors involved histone and nucleosome remodeling, focusing on the posttranslational modification of histone side chain lysine and arginine residues (Fig. 5b), because of their roles in adipogenesis [83–85]. Transcripts encoding the lysine acetyltransferase *KAT2B* and lysine demethylase *KDM4* are expressed at 100- to 1,000-fold higher levels in Uncap-SAT and -VAT nuclei than in the captured fractions. Transcripts for the protein arginine methyltransferase PRMT1 were expressed at 8- to 10-fold higher levels in Uncap-SAT and Uncap-VAT nuclei than in captured. Two other PRMTs, CARM1 (PRMT4) and PRMT5 were 2- to 3-fold more weakly expressed in Uncap-SAT nuclei than in the other three nuclear fractions. Transcripts for *PAXP1* which controls chromatin condensation and gene activation and *ARID1A (BAF250)* a ATP-dependent swi/snf remodeler were 3- to 50-fold more highly expressed in Uncap-SAT and -VAT nuclei than Cap-MSA or Cap-MVA nuclei. In total, the generally higher expression of these transcripts in non-adipocyte nuclei suggest that the non-adipocyte cell types may be turning over the classes of chromatin structures controlled by these remodeling activities faster than adipocytes.

## Discussion

### MAs from SAT and VAT

Mature adipocyte nuclei expressing the SUN1mRFP1Flag reporter were easily captured from SAT and VAT dissected from the MA-INTACT mouse. Most transcript markers of specific for SAT and VAT MAs were expressed at three orders-of-magnitude higher levels in the captured nuclei relative to uncaptured nuclei. This demonstrates not only the exceptional purity of captured nuclei, but also the efficiency of capture, because few nuclei with these activities appear to be left behind in the uncaptured fraction. Similarly, most transcripts specific to leukocyte, endothelial, and progenitor cells were significantly enriched in the uncaptured nuclear fraction. VAT and SAT are relatively distinct in their developmental origins, in their metabolic and gene expression profiles, and in their contribution to obesity related disease [4–10, 86]. So perhaps it is not surprising that a few transcripts considered markers of adipocytes or non-adipocytes were found significantly enriched in the captured MVAs as compared to the uncaptured VAT and/or captured MSA nuclei. We found captured, MVAs expressed significantly higher levels of *TNFA, CEBPA,* and *KLF2* transcripts than MSA, all of which favor reduced levels of adipogenesis in VAT and higher levels of *RETN* and *SERPIN*s, which are linked to obesity-related disease risk. Most of these differences between VAT and SAT have not been reported previously, presumably due to the power of examining mature adipocyte nuclei enriched away from other adipose tissue cell types. As additional explanations for transcripts being unexpectedly enriched in the MVA fractions we suggest: (1) MAs isolated from within their in vivo environment may have heretofore unknown properties distinct from adipocytes matured *in silico* and/or (2) nuclear RNA levels do not represent cytoplasmic RNA levels for a small percent of transcripts [28].

By contrast, both captured and uncaptured nuclei from BAT were slightly but significantly enriched for some classes of marker transcripts, but they expressed the same levels of endogenous *ADIPOQ* transcripts. We anticipated that the *ADIPOQ* promoter driven reporter would not tag thermogenic adipocyte nuclei [61], but in any case, a large percentage of BAT nuclei were captured (Table 2). Our data suggest that expression of the *ADNp::SUNmRFP1Flag* reporter is not cell type specific in BAT. Perhaps endogenous transcription factors and/or transgene position effects alter reporter expression in BAT, problems that do not appear to impact reporter specificity in VAT or SAT.

White adipocyte-derived beige/bright adipocytes within white adipose tissue may behave as thermogenic adipocytes and express PRDM16, a high level transcriptional co-regulator of brown adipocyte development, and the heat generating uncoupling protein UCP1 [21, 87, 88]. However, it was surprising to find the captured MSA and MVA nuclei expressed such exceptionally high levels of nuclear transcripts encoding *PRDM16* and *UCP1,* three orders of magnitude above the levels in non-adipocyte nuclei and above that observed in the adipose tissues themselves and above the endogenous control SDHA. It is unlikely that there were enough of the beige adipocytes enriched in the MSA and MVA nuclear fractions to account for such high transcript levels. Further, ADIPOQ expression is known to suppress UCP1 expression in brown adipocytes [61]. An alternative explanation is that these two transcripts are exceptions to the rule about nuclear transcript levels correlating with cytoplasmic transcripts levels. In the founding paper on INTACT technology [28], there was a strong linear correlation between nuclear and cytoplasmic mRNA levels with an r value of 0.94. In other words, only a small fraction of transcripts do not fit this relationship. Yet, *PRDM16* and *UCP1* transcripts could belong to this subset, and be much more highly expressed in the nucleoplasm than the cytoplasm. If these transcripts remain in the nucleus in most tissue-derived adipocytes, they would not be expressed as protein. If this is the case, then nuclear RNA transport may be an important factor controlling UCP1 and PRDM16 expression in adipocytes. The ongoing transcription and nuclear accumulation of thermogenic transcripts in MAs might create a poised decondensed chromatin state enabling white adipocytes to more rapidly develop into beige adipocytes upon cold induction [89, 90].

### Chromatin remodeling factors in SAT- and VAT-derived adipocytes

Because adipocytes are the defining cell type in adipose tissue, we had considered the following hypothesis: *adipocytes isolated from VAT and SAT express higher levels of transcripts for chromatin remodeling factors than supporting cell types,* perhaps making them more responsive to environmental stimuli. We examined factors involved in DNA cytosine modification and nucleosomal histone side chain modification that could be involved in controlling adipogenesis or maintenance of MAs.

#### DNA cytosine modification in adipose tissue

The development of lipid body-rich MAs proceeds after preadipocytes leave the cell cycle [79] and a complex role for DNA methylation-based gene silencing by has been implicated in this process. Global DNA cytosine methylation levels increase, two days after the differentiation of 3 T3-L1 cells begins [79]. Within the first 24 h of differentiation, expression of the maintenance DNA methyltransferase *DNMT1* increases significantly [91]. Once MAs have developed *DNMT1* levels decrease. The *de novo* cytosine methyltransferase DNMT3A may also play a positive role, because siRNA silencing of *DNMT3A* in 3 T3-LI preadipoctyes significantly blocks adipogenesis [79]. When a cytidine analog and inhibitor of both DNMTs 5-azacytidine (5-azaC) is used to treat bone marrow derived MSCs, a normal precursor of adipocytes, there is a decrease in precursor cell proliferation and adipogenesis [92]. Similarly, treating atrial cardiac cells with 5-azaC leads to the trans-differentiation into lipid body-containing adipocytes [93]. In contrast to these experimental results suggesting a simple positive role for methylation, siRNA silencing of *DNMT1* in preadipocytes accelerates adipogenesis [91]. To understand the role of DNA methylation it is useful to place these results in a larger context. DNA cytosine modification at critical gene region CG dinucleotides proceeds via a complex cycle of methylation to 5mC by DNMTs, oxidation to 5hmC and beyond by TETs, and base excision and repair by TDG, GADD45s, and additional factors. Although steady state levels and gene region distribution of 5mC often appear constant within a cell type, the turnover rate of 5mC has been shown to be quite rapid when measured. Half lives of less than 30 min are not uncommon [94]. Any step in the cycle might be differentially regulated in a particular cell type. Therefore we thought it would be informative to assay representative factors spanning the cytosine modification cycle. Although it is true that we found differential expression of 7 of the 8 factors assayed among one or more of the captured adipocyte nuclei or uncaptured non-adipocyte nuclear fractions, the results do not support our working hypothesis. As particular examples, *TDG, DNMT1, and GADD45A* transcripts are much more highly expressed in uncaptured non-adipocyte SAT nuclei than in MSA nuclei. Only *TET3, TDG,* and *GADD45A* were more highly expressed in MVA nuclei than uncaptured non-adipocyte VAT nuclei fitting the prediction from our hypothesis.

#### Nucleosomal histone modification

We examined the expression of transcripts encoding several nucleosomal histone remodeling factors with presumed roles in adipogenesis or maintenance of adipocytes.

Very early in the differentiation of 3T3-L1 preadipocytes levels of histone 3 lysine 9 methylation (H3K9me3) increase 2- to 3-fold, enabling preadiopcytes to differentiate into MAs [79]. SETDB1 and KDM4 regulate the levels of histone methylation (e.g., H3K9me1, me2, me3). SETDB1 methylates H3K9 and H3K9me1 to H3K9me3, a modification associated with transcriptional repression [95]. PPARg is a high-level regulator of adipogenesis, fatty acid storage and glucose metabolism. Via its modifying activity SETDB1 represses PPARg transactivation at target genes. In opposition to SETDB1, KDM4A/*JMJD2A* is a lysine-specific demethylase that directly demethylates H3K9me3 to H3K9me1/2 [96, 97]. KDM4A is essential to recruiting PPARg to its target genes expressed during adipocyte development [98]. The levels of SETDB1 were not distinct among the nuclear fractions. Somewhat surprising, we found order of magnitude higher levels of KDM4 in the two uncaptured SAT and VAT nuclear populations, relative to levels the captured MSA and MVA fractions. Again these data are contrary to our working hypothesis.

We examined the transcript levels of three histone arginine methyltransferases (PRMTs), *PRMT1, CARM1 (PRMT4)* and *PRMT5*. Among their multiple activities these three PRMTs can methylate four arginine residues on the N-terminus of histone H3 and one on H4 and these modifications are generally associated with gene activation. PRMT4 and PRMT5 appear to play positive roles in adipogenesis by supporting PPARg expression [85, 99–101]. PRMT1 is the dominant type I PRMT and has an essential role in cell proliferation and genome maintenance [102] and defects in PRMT1 are associated with cardiovascular disease [103, 104]. However, specific roles for PRMT1 in preadipoctyes, MAs, or adipogenesis are yet to be described. We found PRMT1 was several fold more highly expressed in VAT and SAT non-adipocyte nuclei, than in captured MSA or MVA nuclei. This may not be surprising considering that PRMT1 appears to be important for the mesenchymal-epithelial transition, which might impact the endothelial cell population. The levels of CARM1 and PRMT5 transcripts fit expectation, at least in part, because their levels were a few-fold higher in captured MSA nuclei than in uncaptured SAT nuclei. ARID1A (BAF250) is the well-studied ATP-dependent swi/snf subunit of several nucleosome remodeling complexes. *ARID1A* transcripts were dramatically more highly expressed in the uncaptured fractions.

In summary, among captured MSA and/or MVA nuclei and uncaptured nuclei, there were distinct differences in the expression levels of 10 of the 16 transcripts examined. However, the results do not support the hypothesis being tested. In fact, for a significant majority of comparisons between captured MSAs and MVAs and their uncaptured counterparts, the uncaptured nuclei expressed higher levels of the chromatin remodelers assayed. Considering the evidence for the high degree of purity of captured MA nuclei, it is unlikely that our results reflect any significant contamination of the captured and uncaptured fractions from SAT or VAT. Although a simple interpretation is not obvious, these results do suggest that the supporting cell types within SAT and VAT are more active in chromatin remodeling than MAs.

## Conclusions

Epigenetics is the study of cell-type specific differences in chromatin structure within an organ or tissue developmental context. To improve cell-type specific analyses of adipocytes we engineered two populations of inbred MA-INTACT mice in which the surface of MA *ADIPOQ* expressing nuclei are tagged. This enabled the efficient immunocapture of MSA and MVA nuclei from within VAT and SAT, separating them from the nuclei of contaminating endothelial, leukocyte, progenitor, and preadipocyte nuclear types. Transcript for classical adipocyte markers were enriched in the captured nuclei by several orders of magnitude, confirming the high levels of purity of MSA and MVA nuclei. Captured and uncaptured nuclei, however, had some novel and unexpected properties. For example, MSA and MVA nuclei expressed high levels of transcripts for two markers of thermogenic beige or brown adipocytes. In addition, the uncaptured populations often expressed higher levels of transcripts encoding chromatin remodeling factors assayed, relative to captured adipocyte nuclei. These data do not support our working hypothesis that adipocytes would be the most epigenetically active cell type in adipose tissue.

MA-INTACT mice may be used to monitor epigenomic, transcriptomic, and proteomic changes in isolated MA nuclei from VAT and SAT and perhaps other tissues that we have not yet examined such as adipocytes within bone and muscle. Our long-term goal was to enable studies on the impact of various environmental influences such as aging, diet, exercise, stress, and drug treatment on adipocytes. The reporter transgene *ADNp::SUN1mRFP1Flag* could be recombined with model mice designed to examine obesity-related diseases in which adipocytes may play direct or indirect roles such as metabolic syndrome, cardiovascular disease, some cancers, Alzheimer’s, and diabetes.

The D line of MA-INTACT mice, homozygous for the *ADNp::SUN1mRFP1Flag* transgene (Additional file 7: Figure S8D) and will be made available for distribution after publication of this manuscript.

## Abbreviations

ADIPOQ, adiponectin; APLN, Apelin; ARID1A (BAF250, SWI-SNF-related AT Rich Interactive Domain 1A); CARM1, PRMT4, Coactivator-Associated Arginine Methyltransferase 1; CEBPA, CCAAT/Enhancer Binding Protein (C/EBP), Alpha; CD144/CDH5, VCAD, Cadherin 5; DNMT1 and DNMT3A, DNA Cytosine-5´-Methyltransferase 1 and 3A; EDNRB, Endothelin receptor type B; ET1, ET-1, Endothelin 1; FABP4, adipocyte protein 2, aP2, FABP4, fatty acid binding protein 4; FAM132A, Adpl, Adipolin, Adipose-Derived Insulin-Sensitizing Factor; GADD45A B, Growth Arrest And DNA-Damage-Inducible, Alpha, Beta; GATA2, endothelial transcription factor GATA Binding Protein 2; IKZF1, Ikaros family zinc finger protein 1; KAT2C, MLL3, KMT2C, Lysine-Specific Methyltransferase 2C; KDM4A, H3K9/36me3 lysine-specific demethylases 4A, JMJD2A Jumonji Domain Containing 2A; IHH, Indian Hedgehog Homolog), IKZF1 (IKAROS family zinc finger protein 1; ILF3, interleukin enhancing binding factor 3; KLF2, zinc finger transcription factor Kruppel-Like Factor 2; LEP, leptin, obesity factor, obesity homolog; SERPINE1, PAI1, Plasminogen activator inhibitor-1; PCAM1, Platelet/Endothelial Cell Adhesion Molecule 1; PPARg2, adipocyte specific 2^nd^ isoform of peroxisome proliferator-activated receptor gamma; PRDM16, PR domain containing 16; PRMT1 and PRMT5, protein arginine methyltransferase 4 and 5; PAXIP1/PTIP, PAX Interacting with Transcription-Activation Domain Protein 1, promotes histone H3 lysine 4 methylation; RETN, Resistin, C/EBP-Epsilon-Regulated Myeloid-Specific Secreted Cysteine-Rich Protein; SDHA, Succinate dehydrogenase complex subunit A; SERPINE1, PAI1, Endothelial Plasminogen Activator Inhibitor; SERPINF1, PEDF, Pigment epithelium-derived factor; SETDB1/KMT1E, SET Domain Bifurcated 1, Histone H3-K9 Methyltransferase 4; SREBF1, sterol regulatory element binding transcription factor 1; TDG, G/T Mismatch-Specific Thymine DNA Glycosylase; TET1, 2, 3, Ten-Eleven Translocation-1, 2, 3, Tet Methylcytosine Dioxygenase, TNFA, tumor necrosis factor alpha, UCP1, uncoupling protein 1; VEGFA, Vascular Endothelial Growth Factor A; ADSC, adipose tissue derived stem cell; DFAT, dedifferentiated adipocyte-derived progeny cell; INTACT, Isolation of Nuclei Tagged in specific Cell Types; MA, mature adipocyte; VAT, SAT, BAT, visceral, subcutaneous, and brown adipose tissue, respectively; MVAs MSAs MBAs, mature adipocytes from VAT, SAT, and BAT, respectively; qRT-PCR, quantitative real-time polymerase chain reaction amplification of cDNA

## Additional files

Additional file 1: Figure S1. Structure of the ADIPOQ expression vector pADNpcDNA3.1 KanR. The indicated multilinker contains a *Bsp*LU11l site (5´-ACATGT) containing the ATG initiation codon and the down stream *Nhe*I site forming the replacement region used in the molecular cloning of five reporter protein coding sequences. (PDF 1382 kb) Additional file 2: Figure S2. Structure of the ADNp::mRFP1 reporter vector in which the CDS of *mRFP1* was cloned into the *BspLU11l* – *Nhe*I replacement region of pADNpcDNA3.1. This reporter was used to test the specificity of expression from the *ADIPOQ* expression cassette. The untethered mRFP1 is expressed in the cytoplasm. (PDF 1382 kb) Additional file 3: Figure S3. Structure of the INTACT reporter vector pADNp::SUN1mRFP1Flag. The *mRFP1Flag* CDS was inserted into the *BspLU11l* – *Nhe*I replacement region of pADNpcDNA3.1. This was the most powerful reporter construct and was used in most applications described in the manuscript. (PDF 1382 kb) Additional file 4: Figure S4. Structure of the INTACT reporter ADNp::mRFP1Nesp. The *mRFP1Nesp* CDS was inserted into the *BspLU11l* – *Nhe*I replacement region of pADNpcDNA3.1. This construct was set aside, after initial screening in 3 T3-L1 derived adipocytes. (PDF 1382 kb) Additional file 5: Figure S5. Structure of the INTACT reporter ADNp::NespmRFP1Nesp. The *NespmRFP1Nesp* CDS was inserted into the *BspLU11l* – *Nhe*I replacement region pADNpcDNA3.1. This construct was set aside, after initial screening in 3 T3-L1 derived adipocytes. (PDF 1382 kb) Additional file 6: Figure S6. Structure of the INTACT reporter ADNp::RANGAP1GFP-BLRP. The *RANGAP1GFP-BLRP* CDS was inserted into the *BspLU11l* – *Nhe*I replacement region pf pADNpcDNA3.1. BspLU11L and NheI sites were added to the RanGap1-GFP-BLRP gene described in Deal & Henikoff [28] by mutagenic PCR. This construct was set aside, after initial screening in 3 T3-L1 derived adipocytes. (PDF 1382 kb) Additional file 7: Figure S8. Breeding of transgenic mouse lines #0023C (C line) and #0025D (D line) expressing the ADNp::SUN1mRFP1Flag construct. A. Litter Progression from Founder #0023 (C line). B. Litter Progression from Founder #25 (D-line). C & D. Monitoring transgene expression during backrosses of C and D line to WT. Relative Quantity of transgene (RQ) from qPCR screening of the first generation of selfing of F2 generation offspring to obtain homozygous lines for the ADNp::SUN1mRFP1Flag transgene. An mRFP1 gene primer pair was used (mRFP Additional file 9: Table S1). CT values were normalized to qPCR values for actin gene ACTB using the dCT method [105] to obtain a relative quantity of gene copies (1x hets, 2x homo, ~0 WT). D. Notice the constant 2x RQ level of *mRFP1* qPCR product when inbreeding homozygous lines. (PDF 1382 kb) Additional file 8: Figure S7. Enrichment protocol for SAT, VAT, and BAT cellular nuclei images showing relative purity. A. Adipose tissue depots examined. B. Summary of protocol for isolating nuclei prior to capture. C. Image of enriched nuclei from VAT, SAT, and BAT. This is a combined image with DIC in green and DAPI DNA fluorescence in red. D. The protein was extracted from tissue and from enriched nuclear preparations, resolved by PAGE-SDS on a 15 % gel run for 1 hr and 15 min at 20 ma and examined for the levels of nuclear protein histone H3 on an immunoblot (anti-H3 mouse monoclonal ab10799). The signal was developed using HRP-conjugated goat anti-mouse antibody (NA931V) and a ECL kit (RPN2106) for detection. Parallel samples were run for 15 min on a duplicate gel and the protein front was stained for total protein with Coomassie blue to show approximately equal loading among paired samples. (PDF 1382 kb) Additional file 9: Table S1. Oligonucleotide primers used during qRT-PCR of transcripts and qPCR of ADNp::SUN1mRFP1Flag transgene. (PDF 1382 kb) Additional file 10: Figure S9. Expression of cell-type- and function- specific transcripts in MA nuclei captured from BAT. qRT-PCR analysis was performed on cDNA prepared from captured adipocyte nuclei from BAT (MBA) and uncaptured nuclei (UnCap-BAT). Nuclear mRNAs were assayed for transcripts encoding (A-F) Cell-type and function markers. *SDHA* was used as the endogenous control as it was nearly equivalently expressed in captured and uncaptured samples relative to the amount of input cDNA. Bar graphs show the Mean ± SEM with *p* < 0.05 *, *p* < 0.01 **, *p* < 0.001 *** indicated for selected comparisons. (PDF 1382 kb)
