# Supplementary figures and images for: Adipocyte nuclei captured from VAT and SAT
Source: BMC Obes. 2016 Jul 19;3:35. doi: 10.1186/s40608-016-0112-6 (PMC4949929; doi:10.1186/s40608-016-0112-6)

# Figure S1. pADNpcDNA3.1 KanR vector plasmid

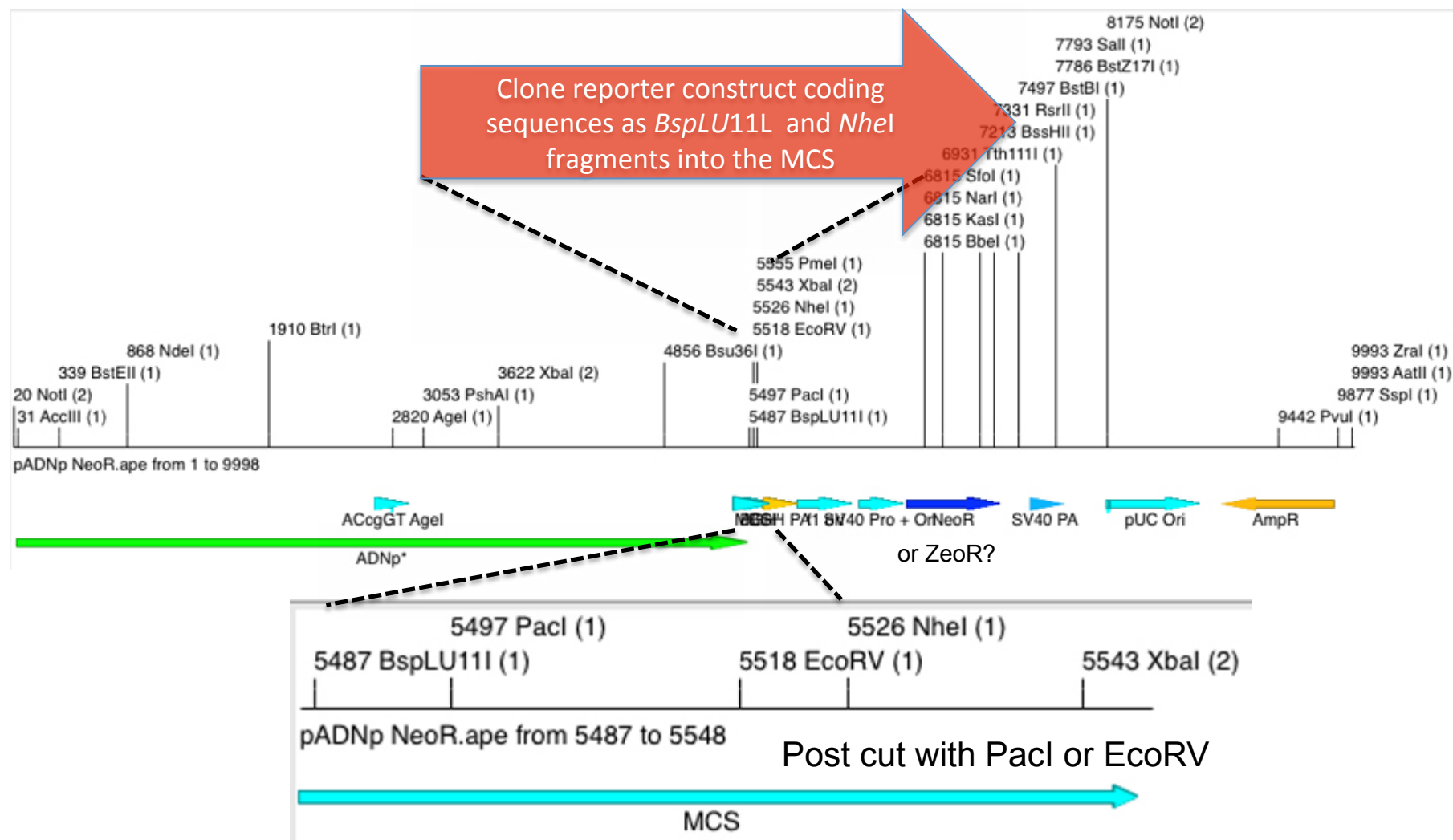

Supplement: Additional file 1: Figure S1. — Structure of the ADIPOQ expression vector pADNpcDNA3.1 KanR. The indicated multilinker contains a BspLU11l site (5´-ACATGT) containing the ATG initiation codon and the down stream NheI site forming the replacement region used in the molecular cloning of five reporter protein coding sequences. (PDF 1382 kb) [file 40608_2016_112_MOESM1_ESM.pdf]

# Figure S2. Map of mRFP1 coding sequence cloned into the expression vector.

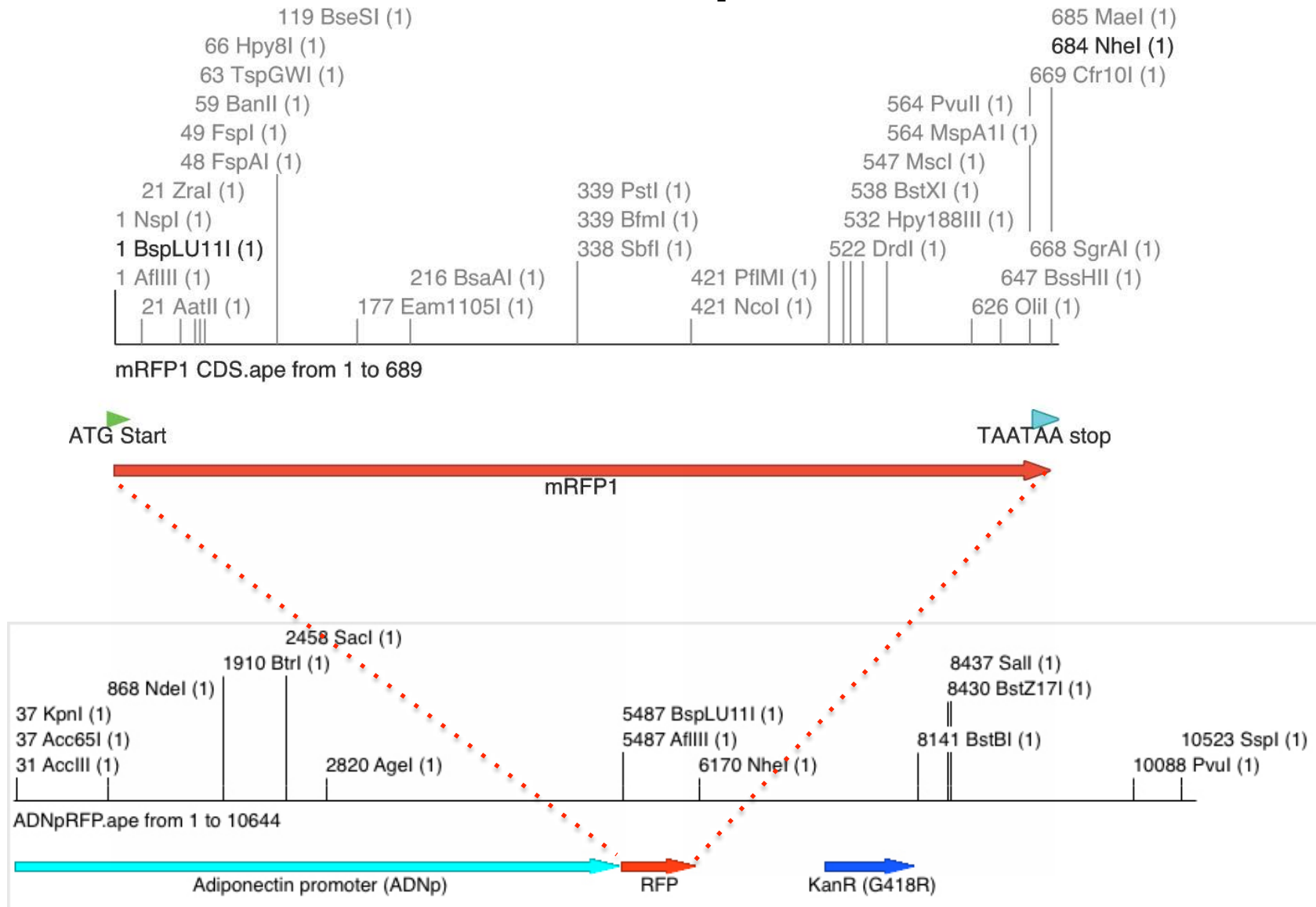

Supplement: Additional file 2: Figure S2. — Structure of the ADNp::mRFP1 reporter vector in which the CDS of mRFP1 was cloned into the BspLU11l – NheI replacement region of pADNpcDNA3.1. This reporter was used to test the specificity of expression from the ADIPOQ expression cassette. The untethered mRFP1 is expressed in the cytoplasm. (PDF 1382 kb) [file 40608_2016_112_MOESM2_ESM.pdf]

# Figure S3. Map of SUN1mRFP1Flag coding sequence

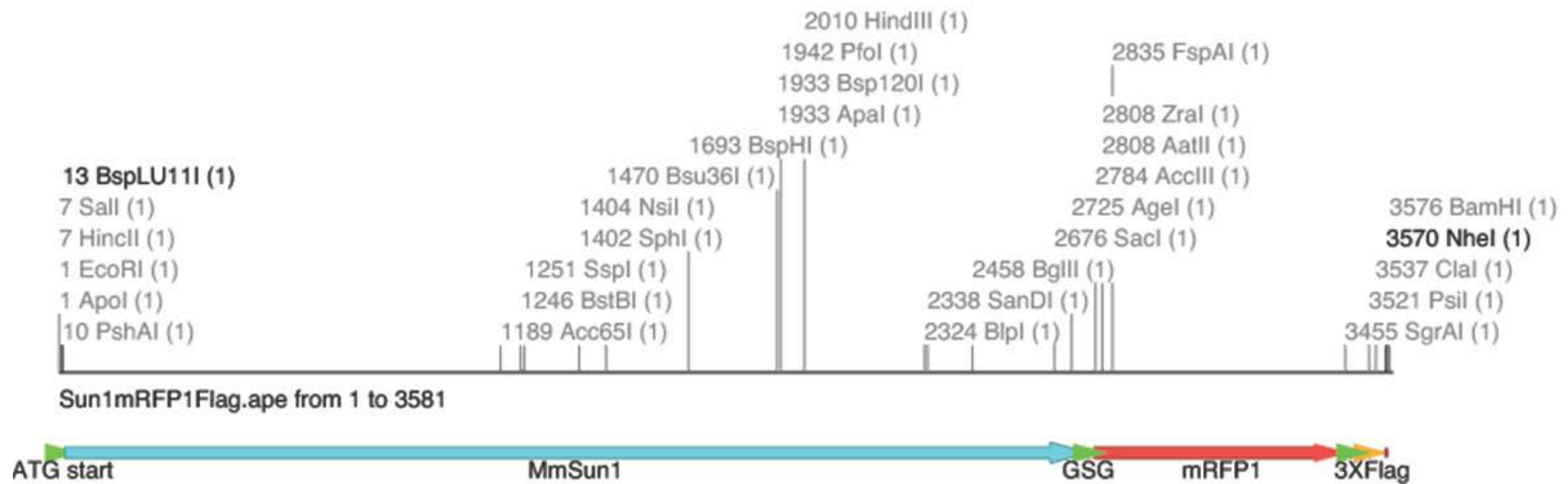

Supplement: Additional file 3: Figure S3. — Structure of the INTACT reporter vector pADNp::SUN1mRFP1Flag. The mRFP1Flag CDS was inserted into the BspLU11l – NheI replacement region of pADNpcDNA3.1. This was the most powerful reporter construct and was used in most applications described in the manuscript. (PDF 1382 kb) [file 40608_2016_112_MOESM3_ESM.pdf]

# Figure S4. Map of Nesp3-mRFP1-Flag coding sequence

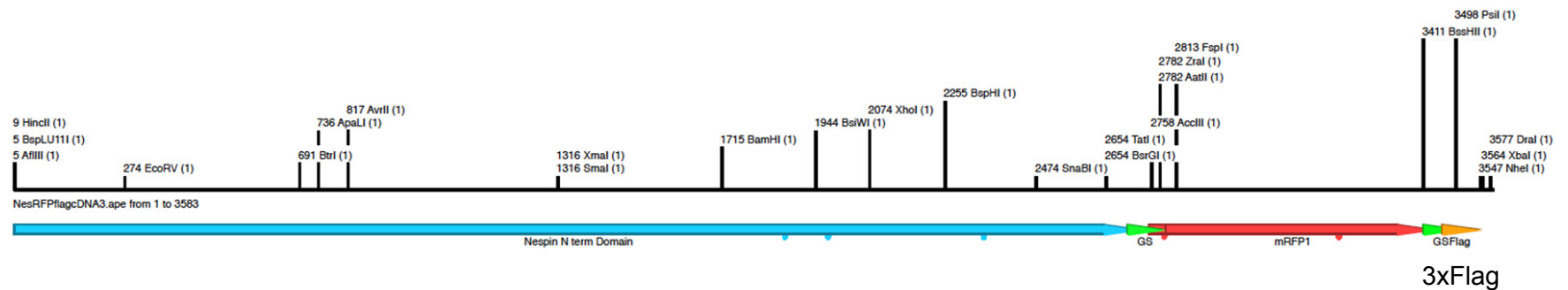

Supplement: Additional file 4: Figure S4. — Structure of the INTACT reporter ADNp::mRFP1Nesp. The mRFP1Nesp CDS was inserted into the BspLU11l – NheI replacement region of pADNpcDNA3.1. This construct was set aside, after initial screening in 3 T3-L1 derived adipocytes. (PDF 1382 kb) [file 40608_2016_112_MOESM4_ESM.pdf]

# Figure S5. Nesp3mRFP1Nesp coding sequence

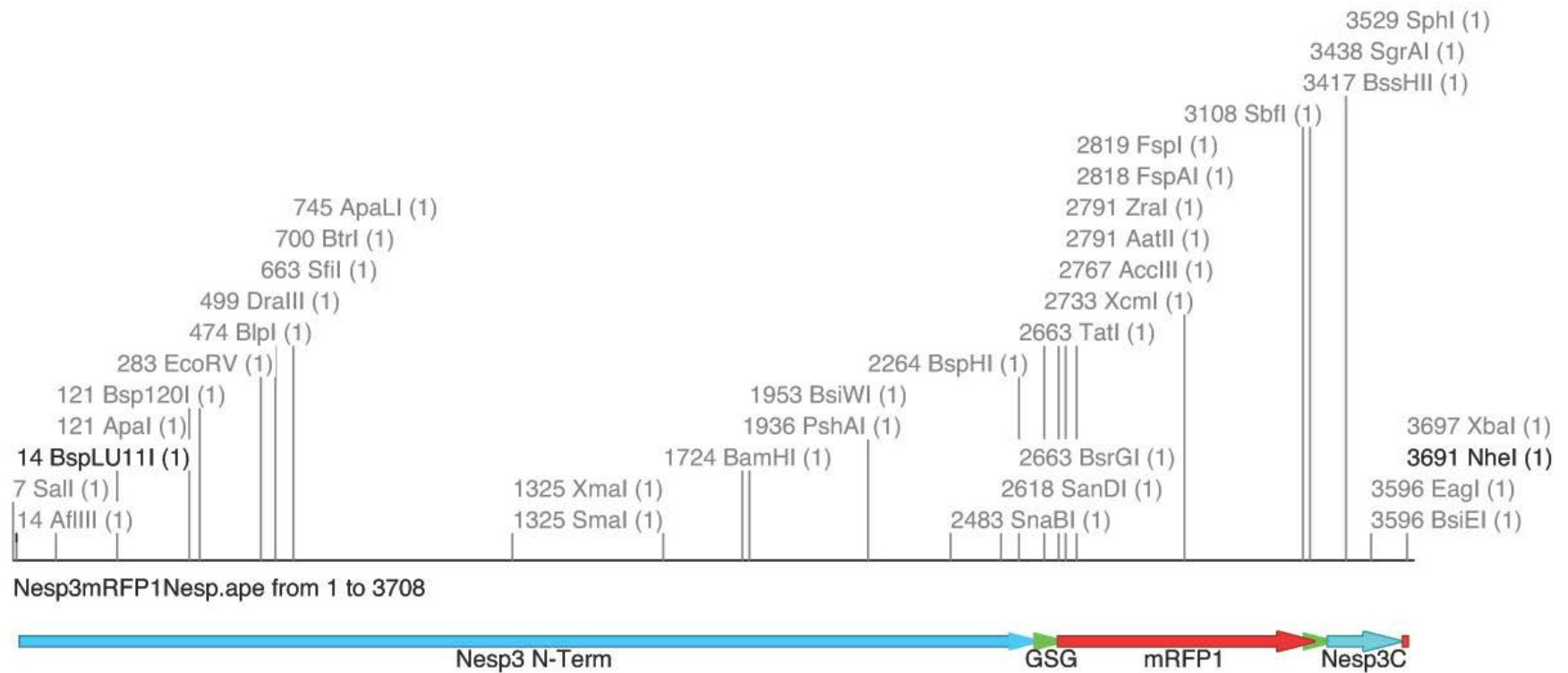

Supplement: Additional file 5: Figure S5. — Structure of the INTACT reporter ADNp::NespmRFP1Nesp. The NespmRFP1Nesp CDS was inserted into the BspLU11l – NheI replacement region pADNpcDNA3.1. This construct was set aside, after initial screening in 3 T3-L1 derived adipocytes. (PDF 1382 kb) [file 40608_2016_112_MOESM5_ESM.pdf]
