# Supplementary material for: Adipocyte nuclei captured from VAT and SAT
Source: BMC Obes. 2016 Jul 19;3:35. doi: 10.1186/s40608-016-0112-6 (PMC4949929; doi:10.1186/s40608-016-0112-6)

Figure S6. Map of RANGAP-GFP-BLRP reporter coding sequence modified from Deal and Henikoff, Dev. Cell, 2010

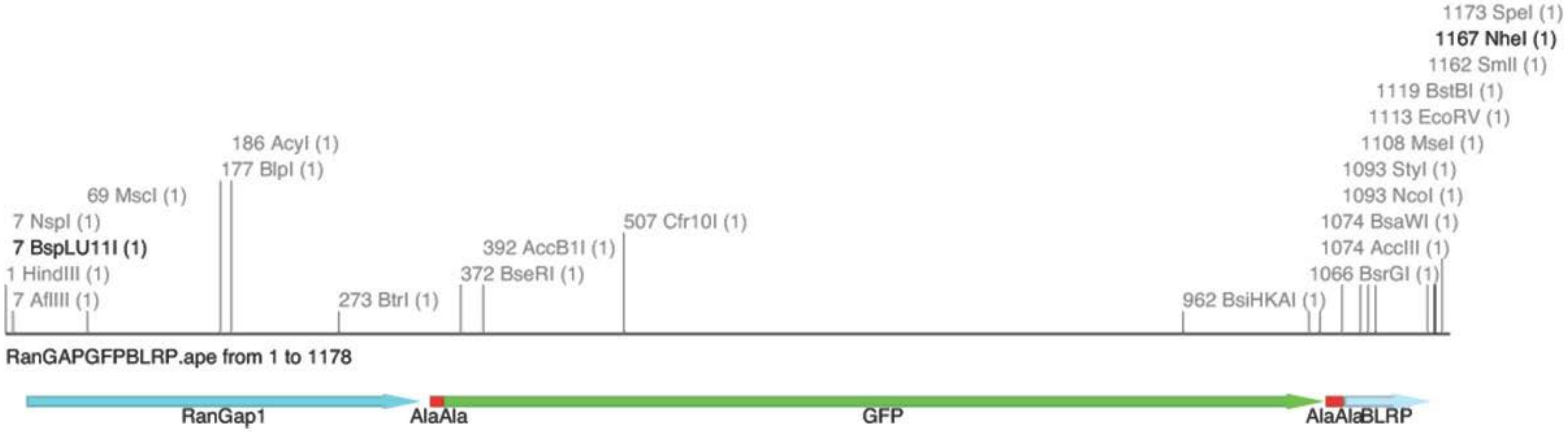

Supplement: Additional file 6: Figure S6. — Structure of the INTACT reporter ADNp::RANGAP1GFP-BLRP. The RANGAP1GFP-BLRP CDS was inserted into the BspLU11l – NheI replacement region pf pADNpcDNA3.1. BspLU11L and NheI sites were added to the RanGap1-GFP-BLRP gene described in Deal & Henikoff [28] by mutagenic PCR. This construct was set aside, after initial screening in 3 T3-L1 derived adipocytes. (PDF 1382 kb) [file 40608_2016_112_MOESM6_ESM.pdf]
