# Supplementary material for: Adipocyte nuclei captured from VAT and SAT
Source: BMC Obes. 2016 Jul 19;3:35. doi: 10.1186/s40608-016-0112-6 (PMC4949929; doi:10.1186/s40608-016-0112-6)
Supplement: Additional file 7: Figure S8. — Breeding of transgenic mouse lines #0023C (C line) and #0025D (D line) expressing the ADNp::SUN1mRFP1Flag construct. A. Litter Progression from Founder #0023 (C line). B. Litter Progression from Founder #25 (D-line). C & D. Monitoring transgene expression during backrosses of C and D line to WT. Relative Quantity of transgene (RQ) from qPCR screening of the first generation of selfing of F2 generation offspring to obtain homozygous lines for the ADNp::SUN1mRFP1Flag transgene. An mRFP1 gene primer pair was used (mRFP Additional file 9: Table S1). CT values were normalized to qPCR values for actin gene ACTB using the dCT method [105] to obtain a relative quantity of gene copies (1x hets, 2x homo, ~0 WT). D. Notice the constant 2x RQ level of mRFP1 qPCR product when inbreeding homozygous lines. (PDF 1382 kb) [file 40608_2016_112_MOESM7_ESM.pdf]

Supplemental Figure S8. Breeding of the ADNp::SUN1mRFP1Flag mice. A. Litter Progression from Founder #23 (C-line)

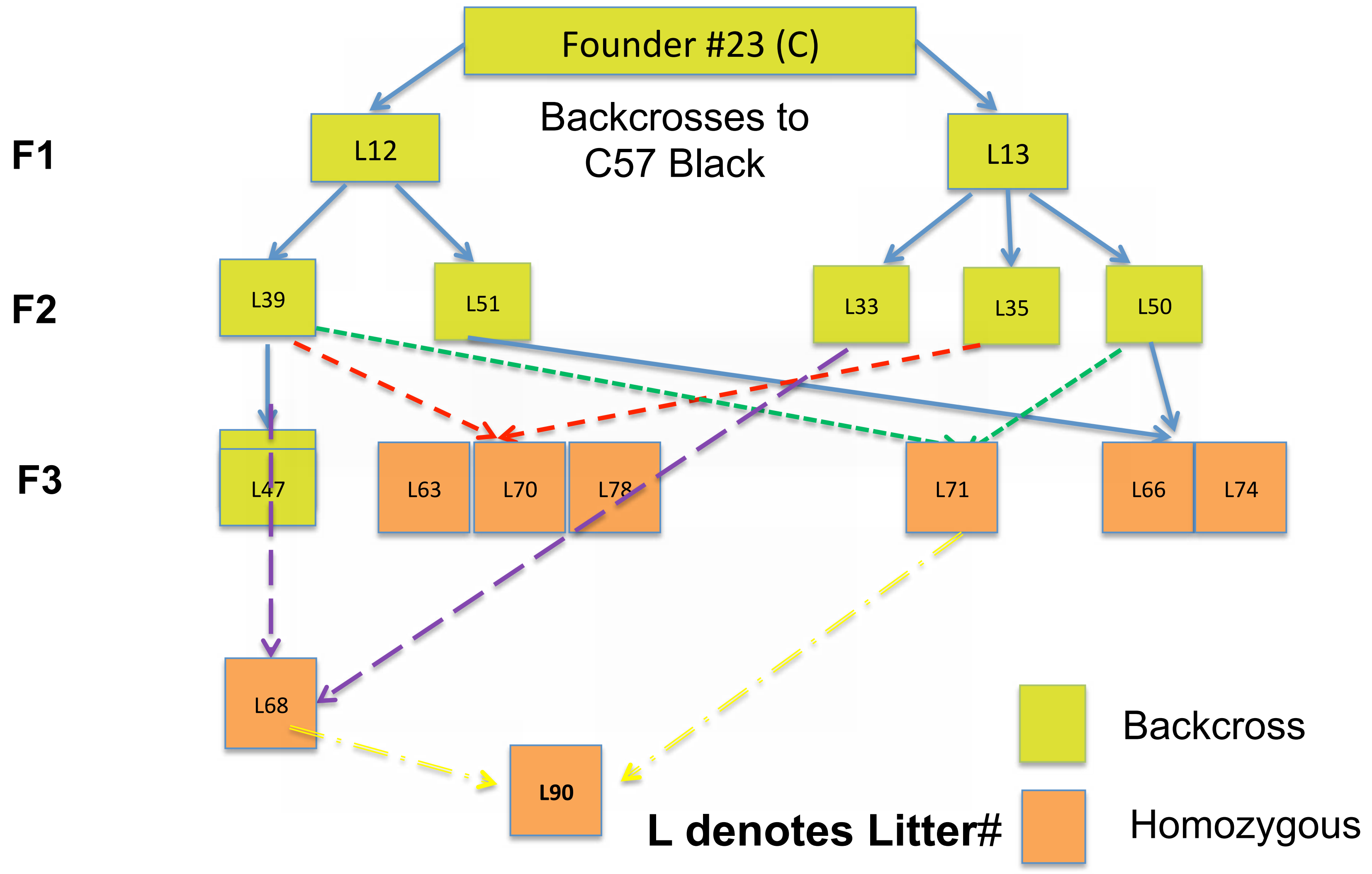

Supplemental Figure S8. C. qPCR analysis of the SUN1mRFP1Flag transgene in D-line during selfing to make homozgyous line.

RQ from qPCR

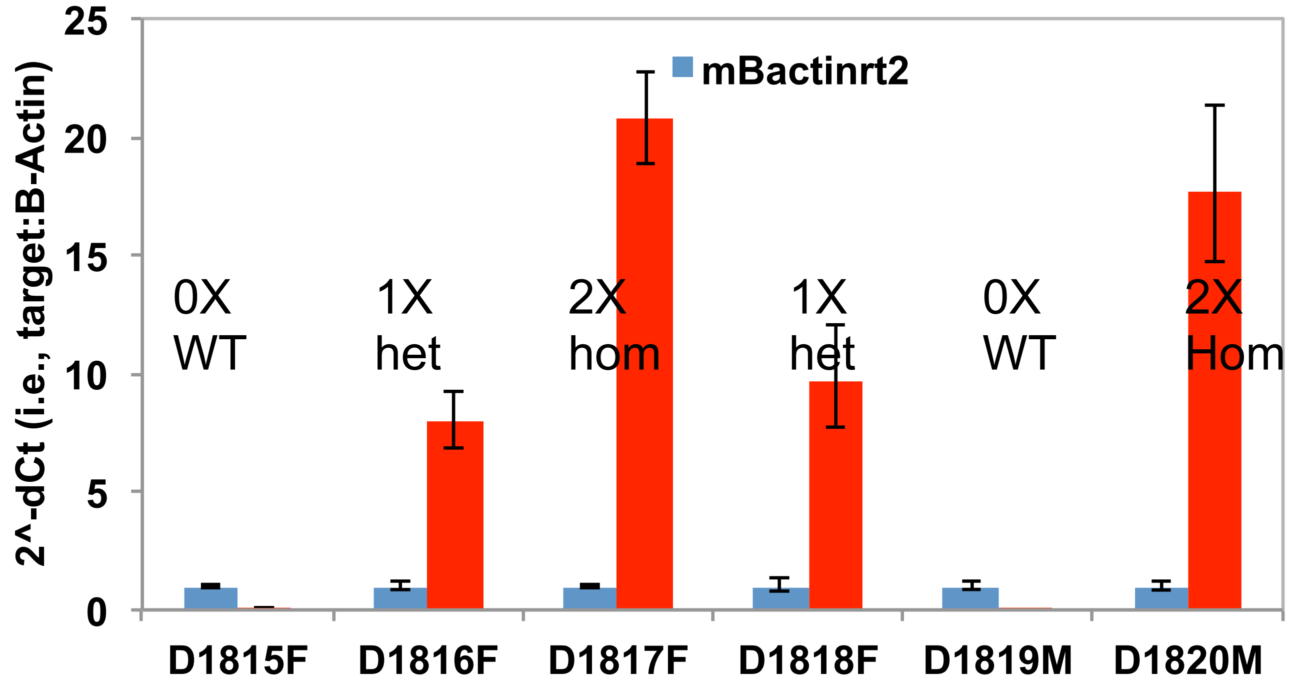

An mRFP1 gene primer pair was used to determine RQ. See Supplemental Table S1.

Supplemental Figure S8. D. qPCR analysis of SUN1mRFP1Flag transgene during maintenance of stable homozygous D-line.

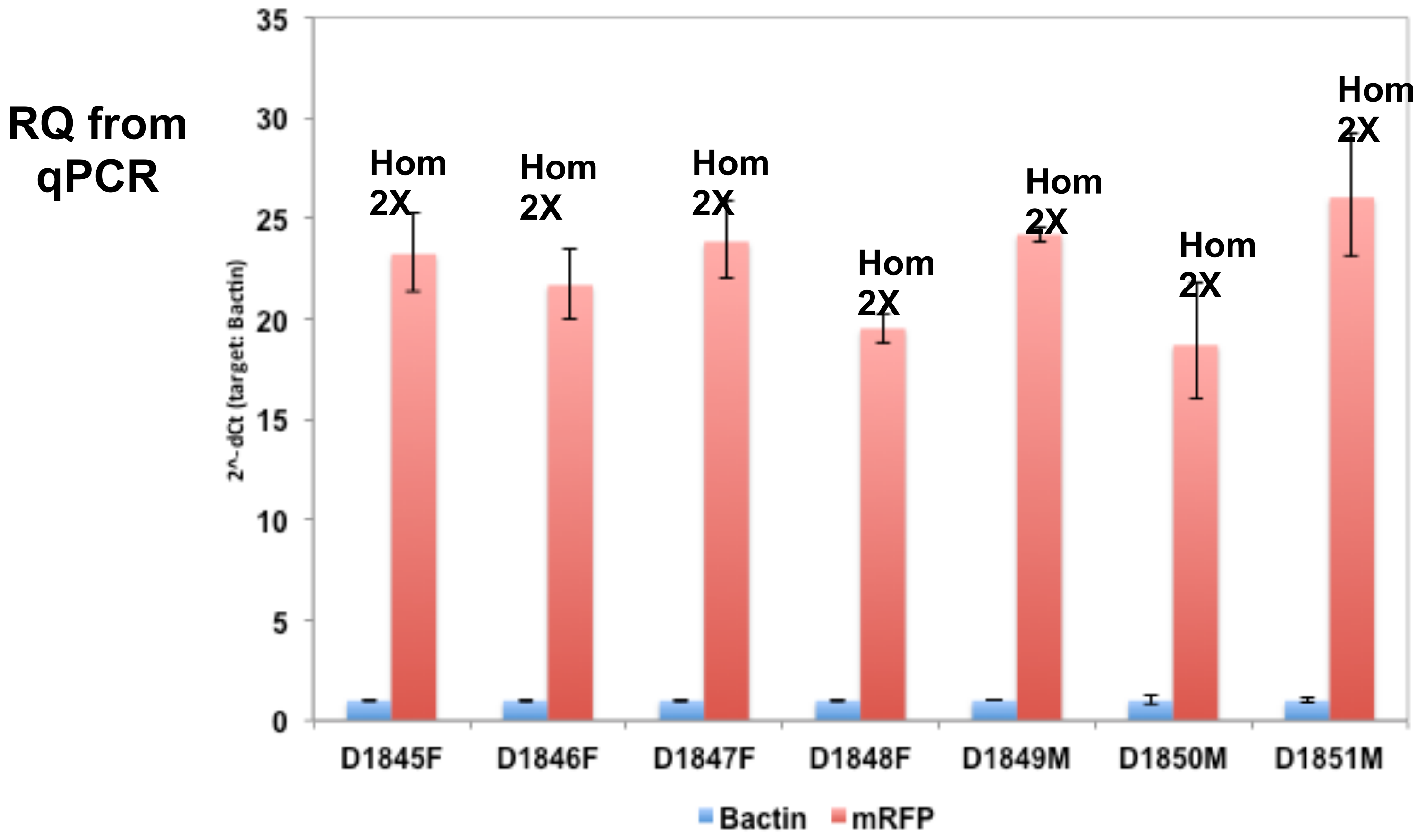

Several dozen mice were screened showing the line is indeed homozygous for the SUN1mRFP1Flag transgene.
