# Supplementary material for: Adipocyte nuclei captured from VAT and SAT
Source: BMC Obes. 2016 Jul 19;3:35. doi: 10.1186/s40608-016-0112-6 (PMC4949929; doi:10.1186/s40608-016-0112-6)

Supplemental Figure S7: Enrichment protocol for SAT, VAT, and BAT cellular nuclei prior to capture.

A. BAT, VAT, and SAT were harvested from 4 locations in the mouse.

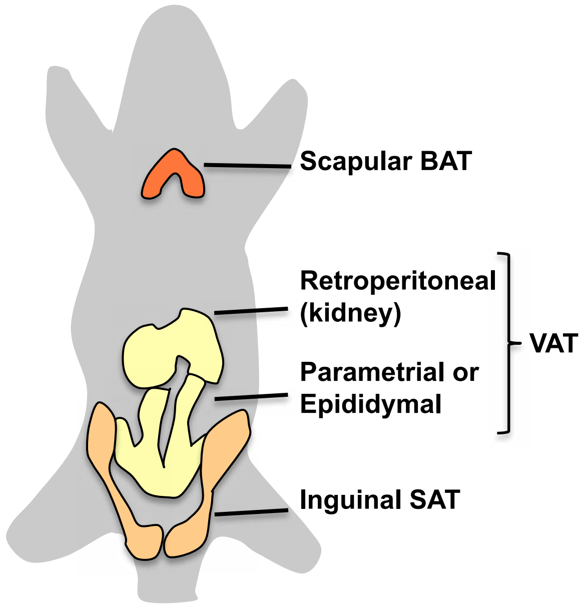

B. Nuclear Isolation Protocol

Fixation (optional)
↓
Homogenize (H)
↓
Miracloth filtration
↓
Sedimentation through sucrose cushion 3,000 x g
↓
Quantify Nuclei (N) in hemocytometer

Supplemental Figure S7: continued

C. Nuclear count & purity of DAPI stained nuclei were evaluated on a hemocytometer using combined IFM and DIC microscopy

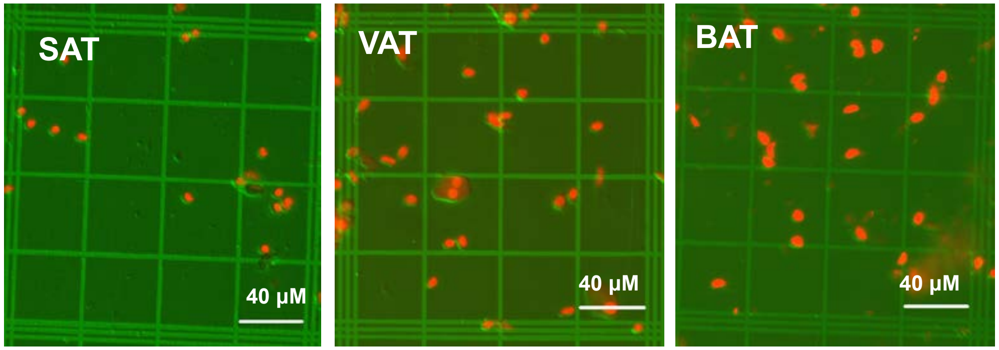

D. Western analysis of nuclear protein enrichment

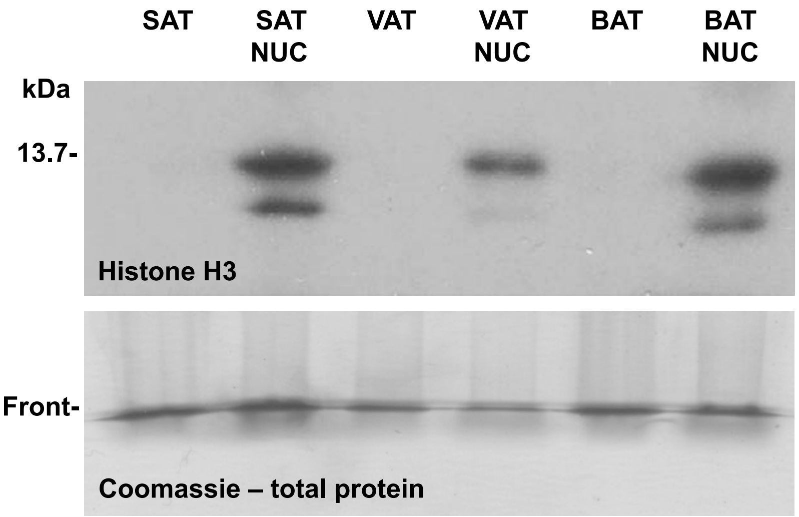

Supplement: Additional file 8: Figure S7. — Enrichment protocol for SAT, VAT, and BAT cellular nuclei images showing relative purity. A. Adipose tissue depots examined. B. Summary of protocol for isolating nuclei prior to capture. C. Image of enriched nuclei from VAT, SAT, and BAT. This is a combined image with DIC in green and DAPI DNA fluorescence in red. D. The protein was extracted from tissue and from enriched nuclear preparations, resolved by PAGE-SDS on a 15 % gel run for 1 hr and 15 min at 20 ma and examined for the levels of nuclear protein histone H3 on an immunoblot (anti-H3 mouse monoclonal ab10799). The signal was developed using HRP-conjugated goat anti-mouse antibody (NA931V) and a ECL kit (RPN2106) for detection. Parallel samples were run for 15 min on a duplicate gel and the protein front was stained for total protein with Coomassie blue to show approximately equal loading among paired samples. (PDF 1382 kb) [file 40608_2016_112_MOESM8_ESM.pdf]
