# Supplementary material for: Adipocyte nuclei captured from VAT and SAT
Source: BMC Obes. 2016 Jul 19;3:35. doi: 10.1186/s40608-016-0112-6 (PMC4949929; doi:10.1186/s40608-016-0112-6)
Supplement: Additional file 9: Table S1. — Oligonucleotide primers used during qRT-PCR of transcripts and qPCR of ADNp::SUN1mRFP1Flag transgene. (PDF 1382 kb) [file 40608_2016_112_MOESM9_ESM.pdf]

Supplemental Table S1. Oligonucleotide primers for qRT-PCR.

Each primer pair listed amplifies a single cDNA based on the derivative of the thermal dissociation curve of each qRT-PCR product using VAT, SAT, and/or BAT cDNA.

Endogenous Controls	Forward	Reverse
Bactin	5´ ACCTTCCAGCAGATGTGGAT 3´	5´ TAGAAGCACTTGCGGTGCACGA 3´
SDHA-S3	5´ CTCTTTCCTACCCGATCACATAC 3´	5´ CCATCTCCAGTTGTCCTCTTC 3´
**Figure 4A**		
ADIPOq	5' CCGGGACTCTACTACTTCTCTT 3'	5' TTCCTGATACTGGTCGTAGGT 3'
PPARg2	5' CAGGCTTCCACTATGGAGTTC 3'	5' GGCAGTTAAGATCACACCTATCA 3'
FABP4	5' GCTCCTCCTCGAAGGTTTAC 3'	5' CCCACTCCCACTTCTTTCAT 3'
LEP	5' TGAGGGTAGAGGATGTGTTAGA 3'	5' GTGGCCACAGAGGTAAGAAA 3'
EDNRB	5´ GGTGGCTGTTCAGTTTCTACT3'	5´ ATCTGCATACCGCTCTTCTTC3´
**Figure 4B**		
IKZF1	5' AAGAGCGATGCCACAACTAC 3'	5' GGACCTCTCTGCTCCTATCTT 3'
PCAM1	5' CACCCATCACTTACCACCTTATG 3'	5' TGTCTCTGGTGGGCTTATCT 3'
FAM132A	5' TGCACCAGGGATCTCACTA 3'	5' CAGCCAGCAGAGCTAACAA 3'
RETN	5' GACAGGAGCTAATACCCAGAAC 3'	5' CAGTTCAGGGACAAGGAAGAA 3'
SERPINF1	5´GGAGTGCAGGATCTCTTTAGC 3´	5´CCTCCTCATTCACTTCCACATAG 3´
TNFα	5' TTGTCTACTCCCAGGTTCTCT 3'	5' GAGGTTGACTTTCTCCTGGTATG 3'
SERPINE1	5' GGGACGAAACTGGAGATGTTAT 3'	5' GGGATGCTGGTTGGAAAGA 3'
CD144	5' CATCTAGGGTTCTGGTCTTTGG 3'	5' CTGGTGTCACTCCTTGGAAAT 3'
ET1	5' GACCATCTGTGTGGCTTCTAC 3'	5' GGAACACCTCAGCCTTTCTT 3'
**Figure 4C**		
ILF3	5' CTTCCCTTCAGATGCCACTAC 3'	5' CATATTTGAGGCCACGTCTCT 3'
**Figure 4D**		
GATA2	5' ATACCCACCTATCCCTCCTATG 3'	5' AGCCTTGCTTCTCTGCTTAG 3'
KLF2	5 'GGCTAGATGCCTTGTGAGAAA 3'	5' TGCCATCGTCTCCCTTATAGA 3'
IHH	5' CCATCACTCAGAGGAGTCTTTAC 3'	5' CGCCAGCAGTCCATACTTATT 3'
VEGFA	5' AGGCTGCTGTAACGATGAAG 3'	5' TCTCCTATGTGCTGGCTTTG 3'
CEBPA	5' GGTTTCCTGGGTGAGTTCAT 3'	5' AACCTAGGTCTCTGTCTCCTAC 3'
**Figure 4E & 4F**		
UCP1	5' CACTGTCTGTCTGGACTTCATC 3'	5' CAAAGGGTTTGTGGCTTCTTT 3'
PRDM16	5' ACCTGCCACAGCAAAGAA 3'	5' CCATCCAAGCAGAGAAGTAGAC 3'
**Figure 5A**		
TET1	5' CTGAAGATGACAAGCAGCAAAC 3'	5' GGTGTGTGTCAGTGGGTAAA 3'
TET2	5' CTCATGCCTCGGGTTCATATT 3'	5' CTGGCACACTCCCATTGTAT 3'
TET3	5' CTTCCCTCCTTTGGCTACTATG 3'	5' GAGGTCTGGCTTCTTCTCAAA 3'
TDG	5' CCACGAATAGCGGTGTTTAATG 3'	5' GAGTTTCTGTGTCTGGGATCTT 3'
DNMT1	5´ CCATCTTCTTGTCTCCCTGTATG 3´	5´ GGTGCTTTGTCCTTCTCCTT 3´
DNMT3A	5´ GTCTCAACAGCACCATTCCT 3´	5´ TGTGGTAGGCACCTGAAATAC 3´
GADD45B	5´ GTGAAGAGAGCAGAGGCAATAA3´	5´ CGACAGTTGCTTTAGATGTTTGG3´
GADD45A	5´ GATGACTTTGCAGAGGGAGAG 3´	5´ CTTTCTTGCAGTGCTTTGTAGTT 3´
**Figure 5B**		
KAT2B	5' AGGAGACCTCCAGCAGATAAT 3'	5' CCATCTCTTCCTCTGACACATTC 3'
KDM4A	5' ATTTGGAGCCTTCAGTCAGAG 3'	5' CAGTGGTGGTAAAGCACATTTC 3'
PRMT1	5' TGGAGGACTACCTAACAGTGAA 3'	5' GAAGTCCAGGTCGATGGTAAAG 3'
CARM1	5' GGCGATTTGCACAGGATAGA 3'	5' GGAGCCAATGAAAGCAACATC 3'
PRMT5	5' GAACTCTGAAGCGGCTATGT 3'	5' CTGGCCAGATTCGTGTTATCT 3'
PAXIP1	5' GCTAACATCCAGCCTTCTTCT 3'	5' ACCCAGTGAAGAGCACTAAAG 3'
ARID1A	5' TATGGAGGTCCTTACGACAGAG 3'	5' GGGTGGAAGGCATGAGATTT 3'
SETDB1	5' CGCCTGGAACCTATGTTTAGT 3'	5' CTCCTCACAGCACCCATATTAG 3'
**Figure S8 A-E**		
mRFP	5' CCGTAATGCAGAAGAAGACCA 3'	5' CTTCAGCTTCAGCCTCATCTT 3'
