# Supplementary material for: Adipocyte nuclei captured from VAT and SAT
Source: BMC Obes. 2016 Jul 19;3:35. doi: 10.1186/s40608-016-0112-6 (PMC4949929; doi:10.1186/s40608-016-0112-6)

Supplemental Figure S9. B. Litter Progression from Founder #25 (D-line)

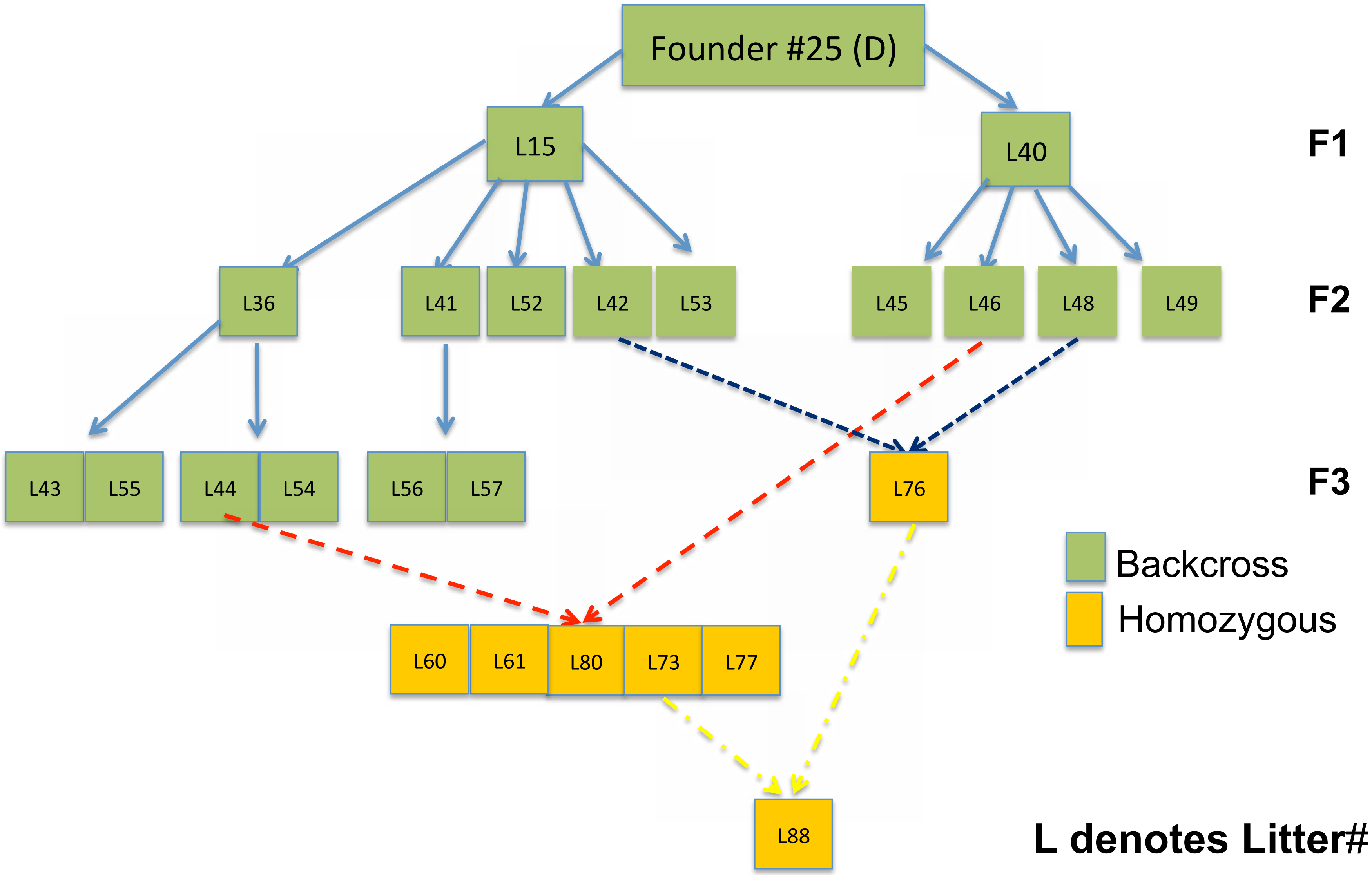

Supplemental Figure S9 (S9A, S9B). Expression of cell-type- and function- specific transcripts in captured and captured nuclei from BAT.

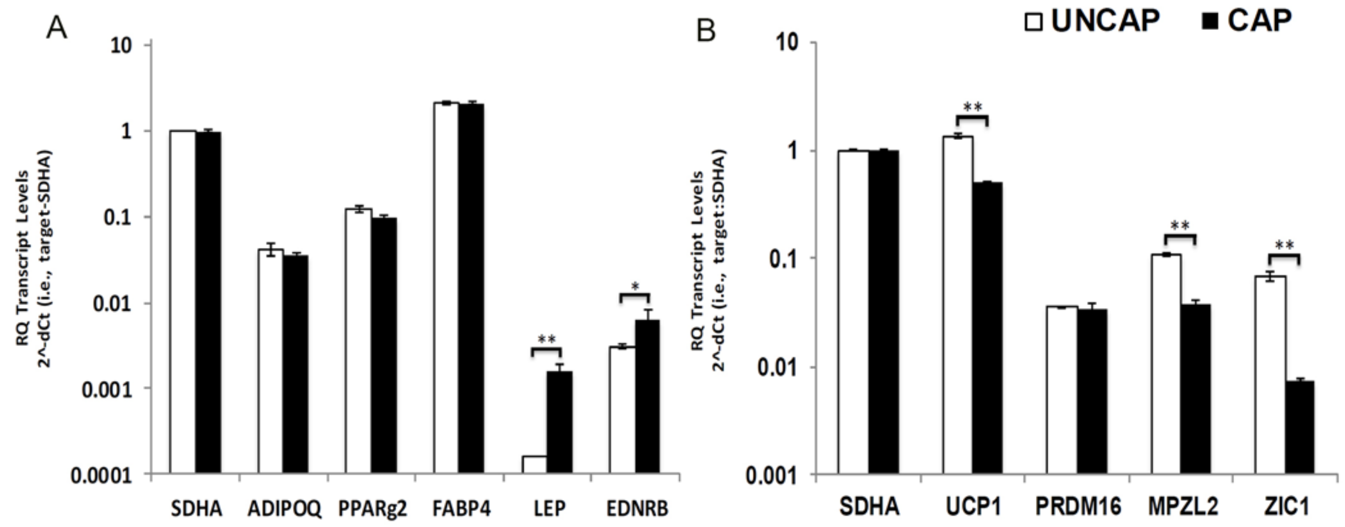

qRT-PCR analysis was performed on cDNA prepared from captured adipocyte nuclei from BAT (CAP) and uncaptured nuclei (UnCap-BAT). Nuclear mRNAs were assayed for transcripts encoding (**A-F)** Cell-type and function markers. *SDHA* was used as the endogenous control as it was nearly equivalently expressed in captured and uncaptured samples relative to the amount of input cDNA. Bar graphs show the Mean ± SEM with $p<0.05$ *, $p<0.01$ **, $p<0.001$ *** indicated for selected comparisons.

Supplemental Figure S9C, D (Continued). Expression of cell-type- and function- specific transcripts in captured and captured nuclei from BAT.

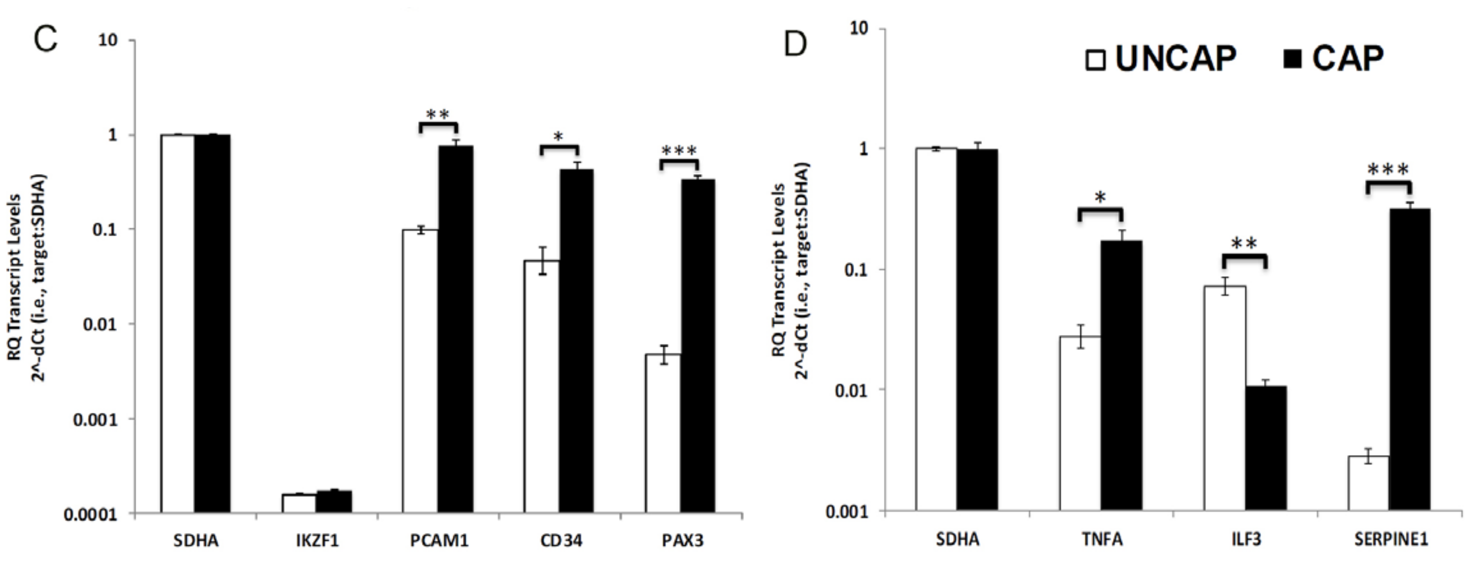

Supplemental Figure S9E, F (Continued). Expression of cell-type- and function- specific transcripts in captured and captured nuclei from BAT.

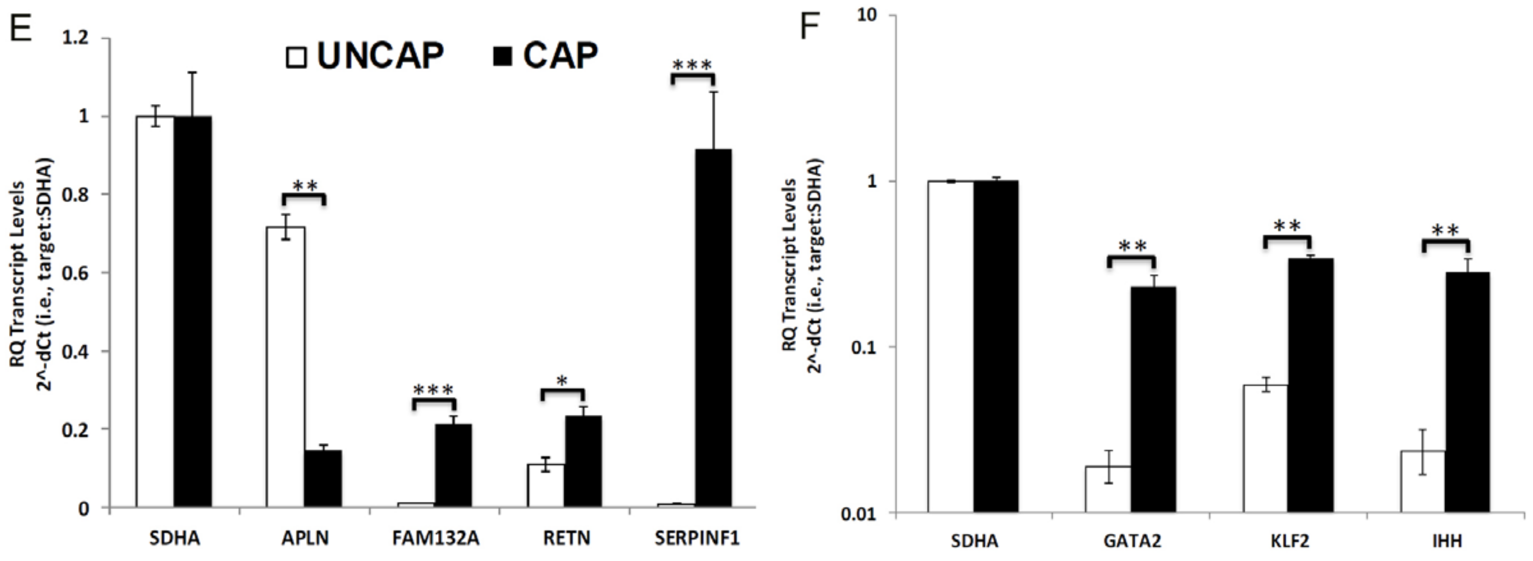

Supplement: Additional file 10: Figure S9. — Expression of cell-type- and function- specific transcripts in MA nuclei captured from BAT. qRT-PCR analysis was performed on cDNA prepared from captured adipocyte nuclei from BAT (MBA) and uncaptured nuclei (UnCap-BAT). Nuclear mRNAs were assayed for transcripts encoding (A-F) Cell-type and function markers. SDHA was used as the endogenous control as it was nearly equivalently expressed in captured and uncaptured samples relative to the amount of input cDNA. Bar graphs show the Mean ± SEM with p < 0.05 *, p < 0.01 **, p < 0.001 *** indicated for selected comparisons. (PDF 1382 kb) [file 40608_2016_112_MOESM10_ESM.pdf]
